# Differential Physiological Prerequisites and Gene Expression Profiles of Conidial Anastomosis Tube and Germ Tube Formation in *Colletotrichum gloeosporioides*

**DOI:** 10.3390/jof7070509

**Published:** 2021-06-25

**Authors:** Nikita Mehta, Ravindra Patil, Abhishek Baghela

**Affiliations:** 1 National Fungal Culture Collection of India (NFCCI), Biodiversity and Palaeobiology Group, MACS-Agharkar Research Institute, G.G. Agarkar Road, Pune 411004, India; nikitamehta@aripune.org; 2 Department of Microbiology, Savitribai Phule Pune University, Pune 411007, India; 3 Genetics and Plant Breeding Group, MACS-Agharkar Research Institute, G.G. Agarkar Road, Pune 411004, India; rmpatil@aripune.org

**Keywords:** *Colletotrichum gloeosporioides*, germ tube, conidial anastomosis tube, transcriptome, qPCR, anthracnose, gene annotation

## Abstract

The conidia of a hemibiotrophic fungus, *Colletotrichum gloeosporioides*, can conventionally form a germ tube (GT) and develop into a fungal colony. Under certain conditions, they tend to get connected through a conidial anastomosis tube (CAT) to share the nutrients. CAT fusion is believed to be responsible for the generation of genetic variations in few asexual fungi, which appears problematic for effective fungal disease management. The physiological and molecular requirements underlying the GT formation versus CAT fusion remained underexplored. In the present study, we have deciphered the physiological prerequisites for GT formation versus CAT fusion in *C. gloeosporioides*. GT formation occurred at a high frequency in the presence of nutrients, while CAT fusion was found to be higher in the absence of nutrients. Younger conidia were found to form GT efficiently, while older conidia preferentially formed CAT. Whole transcriptome analysis of GT and CAT revealed highly differential gene expression profiles, wherein 11,050 and 9786 genes were differentially expressed during GT formation and CAT fusion, respectively. A total of 1567 effector candidates were identified; out of them, 102 and 100 were uniquely expressed during GT formation and CAT fusion, respectively. Genes coding for cell wall degrading enzymes, germination, hyphal growth, host-fungus interaction, and virulence were highly upregulated during GT formation. Meanwhile, genes involved in stress response, cell wall remodeling, membrane transport, cytoskeleton, cell cycle, and cell rescue were highly upregulated during CAT fusion. To conclude, the GT formation and CAT fusion were found to be mutually exclusive processes, requiring differential physiological conditions and sets of DEGs in *C. gloeosporioides*. This study will help in understanding the basic CAT biology in emerging fungal model species of the genus *Colletotrichum*.

## 1. Introduction

The asexual spore or conidium is essential in the life cycle of many fungi because it is the key for dispersal and serves as a safehouse for the fungi in unfavorable environmental conditions. Under favorable conditions, the conidia germinate to form hyphae and finally a fully grown fungal colony. The mechanics of conidial germination are diverse and vary between different fungal species [1]. In general, the first morphological change in conidial germination is isotropic growth, also called the swelling of conidium. The next stage is polarized growth that results in forming a germ tube (GT) that extends and successively branches to establish the fungal colony [2]. However, under certain conditions such as starvation, a conidium will form specialized hypha, called conidial anastomosis tubes (CATs), instead of forming GTs. The CAT fusion between conidia results in an interconnected germling network, allowing genetic exchange and the sharing of nutrients, water, and cell organelles between conidia [3], which has been theorized to improve survival chances in an adverse environment. 

The CAT has shown to be morphologically and physiologically distinct from GT in *Colletotrichum lindemuthianum*, *Neurospora crassa,* and *Fusarium oxysporum* [4,5,6]. In general, CATs are short, thin, and usually unbranched compare to GTs. CATs grow towards each other, while GTs avoid each other [7]. It has also been shown that GT formation and CAT fusion are antagonistic in *Colletotrichum fructicola, Colletotrichum nymphaeae*, and *Colletotrichum theobromicola* [8]. The CAT fusion versus GT formation were independent of conidial age in *N. crassa* and *F. oxysporum* [4,5,9]. Interestingly, older conidia were optimal for CAT fusion while younger conidia were suitable for GT formation in *C. lindemuthianum, C. gloeosporioides,* and *C. siamense* [10,11]. The CAT fusion might play a vital role in the life cycle of fungi by improving the chances of colony establishment during nutrient starvation [7]. However, there are contrasting reports on the relationship between CAT induction and nutrient availability. CAT fusion is inhibited in high nutrient conditions in *C. lindemuthianum* and *C. graminicola*, which only occurs in water/water agar media [6,12]. In *N. crassa* and *F. oxysporum*, CAT induction requires some nutrients to occur [4,5]. Therefore, it is not clear how conidial age and the availability of nutrients can influence the conidial propensity to either undergo CAT fusion or GT formation. 

CAT fusion was shown to play a role in the generation of phenotypic and genetic variations in few fungi. The heterokaryotic progenies have been generated through CAT fusion with distinct phenotypes compared to parental strains in *F. oxysporum* and *C. lindemuthianum* [9,13]. Inter-specific CAT fusion between *C. lindemuthianum* and *C. gossypii* resulted in the formation of some hybrids, which exhibited phenotypic variations; one hybrid was highly pathogenic [14] Furthermore, we have demonstrated that an inter-specific CAT fusion between *C. gloeosporioides* and *C. siamense* generated genetic and phenotypic diversity in these fungal pathogens [11]. Pathogen populations with higher genetic variations tend to show increased mean fitness, resilience, expansion of host range, and fungicide resistance. Therefore, it is imperative to decipher the unique and specific physiological and molecular requirements for CAT fusion and GT formation, which will add to the understanding of the basic biology of CAT fusion.

*C. gloeosporioides* starts its usual life cycle from conidial germination to the formation of melanized infection structures, appressoria [15]. The formation of GTs and CATs in *C. gloeosporioides* were reported in both in vitro and in vivo conditions [11,16]. Despite having legitimate morphological variations between GT and CAT, their whole transcriptome comparison has never been reported. Therefore, *C. gloeosporioides* can be considered as an alternative model organism and as a proxy to understand the underlying physiological and molecular requirements for GT formation and CAT fusion. In the present study, we systematically investigated the in vitro dynamics and the nutritional and physiological requirements of GT formation and CAT fusion in *C. gloeosporioides*. Furthermore, a comparative transcriptome analysis of CAT fusion and GT formation was done to decipher the underlying differential molecular prerequisites for these two processes in *C. gloeosporioides*. 

## 2. Materials and Methods

### 2.1. In Vitro Dynamics of GT Formation and CAT Fusion 

To study the effect of conidial age on GT formation and CAT fusion and their mutual exclusiveness in *C. gloeosporioides*, the GT and CAT induction assays were carried out by following a protocol developed in our lab [11]. The *C. gloeosporioides* (CBS 953.97) culture was inoculated on bean pod agar medium (autoclaved French bean pods submerged in 2% water agar) and was incubated in the dark at 25 °C to induce conidiation. Post-inoculation, the conidia were harvested at different time points, viz., 6, 10, 13, and 17 days. Conidial cells were counted using Neubauer’s chamber and conidial suspension of 4 × 10^5^ per ml was prepared in distilled water and potato dextrose broth (PDB) for CAT and GT induction, respectively. One ml of such conidial suspensions was placed in a 24-well tissue culture plate (Tarsons, Kolkata, West Bengal, India) and incubated in the dark at 25 °C for different time points, viz., 3, 6, 12, 18, and 24 h for GT formation and 24, 48, 72, and 96 h for CAT fusion. They were then examined under a microscope (Olympus BX53 with Olympus DP73 camera, Cellsens 1.13 imaging software) using differential interference contrast (DIC) optics. The GT formation and CAT fusion were quantified as the percentage of conidia (*n* = 300) involved in GT formation or CAT fusion [6]. Different stages of GT formation such as initiation, adhesion, and elongation, and various stages of CAT formation such as induction, homing, and fusion were examined at different time points. 

Different conidial numbers were tested to determine the threshold of conidial concentrations required for CAT fusion and GT formation in *C. gloeosporioides.* Different concentrations of 6-day-old conidia were incubated in PDB for 18 h in the dark for GT induction. In contrast, for CAT induction, different concentrations of 17-day-old conidia were incubated in dH_2_O for 72 h in the dark. The concentrations of conidia tested were 4 × 10^2^, 4 × 10^3^, 4 × 10^4^, 4 × 10^5^, and 4 × 10^6^ per mL [11].

### 2.2. Effects of Availability of Nutrients on GT and CAT Induction

To study whether CAT fusion and GT formation in *C. gloeosporioides* are dependent on the availability of nutrients and starvation stress conditions, CAT fusion (in 17-day-old conidia) and GT formation (in 6-day-old conidia) were induced in nutrient-limiting conditions (water), and high nutrient conditions such as PDB, 2% glucose. Furthermore, to study whether the CAT induction or GT formation in *C. gloeosporioides* is mediated through MAP kinase (MEK) pathway, CAT fusion and GT formation percentage was also determined in the presence of an MEK inhibitor InSolution^™^ PD98059 (5 µM).

### 2.3. Whole Transcriptome Analysis of Resting Conidia, GT and CAT

#### 2.3.1. RNA Extraction, Quantification, and Qualification 

GT formation was induced in 6-day-old conidia at 4 × 10^5^ conidia/mL density in PDB incubated for 18 h in the dark, and the CAT induction was carried out in 17-day-old conidia at 4 × 10^5^ conidia/mL density in water incubated for 72 h in the dark. After that, total RNA was isolated from resting conidia (6-day-old conidia grown on bean pod agar media), germinating conidia (GTs), and fused conidia via CAT (CATs) (3 replicates of each sample) of *C. gloeosporioides* using NucleoSpin RNA Plant kit (Macherey-Nagel, Düren, Germany) according to manufacturer’s guidelines. DNAse treatment was carried out using the Amplification grade DNase I kit (Sigma Aldrich, St. Louis, MI, USA). Isolated total RNA was quantified and qualified using a Nanodrop ND-1000 spectrophotometer and Qubit fluorometer. RNA quality was also assessed using formaldehyde agarose gel electrophoresis. RNA integrity was checked using a Bioanalyzer chip and Agilent RNA TapeStation (Agilent Technologies, Santa Clara, CA, USA).

#### 2.3.2. Library Preparation and Transcriptome Sequencing 

Three µg of total RNA from the three technical replicates of each life stage, namely, resting conidia, GTs, and CATs of *C. gloeosporioides,* were pooled, and independent library preparations were carried out for these three different life stages. Pooling of the RNA samples for library preparation was done due to economic constraints. RNA-seq library preparation was performed as per Illumina-compatible NEBNext UltraTM Directional RNA Library Prep. Sequencing for 150 bp length paired-end (PE) reads was performed in an Illumina HiSeq sequencer.

#### 2.3.3. Quality Control, De Novo Assembly, and Sequence Clustering 

Reads were processed for quality assessment and low-quality filtering before the assembly. The raw data generated was checked for the quality using FastQC, and preprocessing of the data, which includes removing the adapter sequences and low-quality bases (<q30), was done with Cutadapt [17].

Processed reads were assembled using a graph-based approach by the rnaSPAdes program [18]. rnaSPAdes is a tool for de novo transcriptome assembly from RNA-Seq data and is suitable for all kinds of organisms. First, the characteristic properties, including N50 length, average length, maximum length, and the minimum length of the assembled contigs, were calculated. Then, in the second step of the assembly procedure, clustering of the assembled transcripts based on sequence similarity is performed using CD-HIT-EST [19] with 95% similarity between the sequences, which reduces the redundancy without exclusion of sequence diversity that is used for further transcript annotation and differential expression analysis.

#### 2.3.4. Read Mapping to the Reference Genome and Differential Counting

To assess the quality of the assembly, an evaluation of the read content approach was used. Processed reads from all three libraries were aligned back to the final assembly using Bowtie2 [20] with end-to-end parameters. *C. gloeosporioides* Nara gc5 isolate (ftp://ftp.ncbi.nlm.nih.gov/genomes/genbank/fungi/Colletotrichum_gloeosporioides/ (accessed on 8 April 2019) was used as a reference genome. For each sample, the count of mapped reads was derived and normalized to RPKM (reads per kilobase of exon model per million mapped reads). The count data was further used to identify differentially expressed genes at each time point using the DESeq package [21]. Sequencing (uneven library size/depth (accessed on 8 April 2019) bias among the samples was removed by library normalization using size factor calculation in DESeq. Fold changes were determined according to the formula “Expression of treated sample/Expression of control sample”. The raw *P* values were adjusted for multiple testing with the procedure described by [22] to control the FDR (false discovery rate). Finally, the regulation for each transcript was assigned based on their log2fold change.

#### 2.3.5. Gene Ontology Enrichment, Differential Expression Analysis, and Functional Annotation

Assembled transcripts were functionally enriched and categorized based on blast sequence homologies and gene ontology (GO) annotations describing biological processes and molecular functions using Blast2GO software (*p* < 0.05), selecting the NCBI blast Fungi as a taxonomy filter and the default parameters [23]. Out of 121,260 total, 88,491 transcripts (72%) were functionally annotated against all fungal protein sequences from the Uniprot Protein database. Those transcripts with more than 30% identity as a cutoff were taken for further analysis. 

Multiple databases (Uniprot, NCBI, KEGG pathway, and Pfam) were used to annotate the transcripts and determine the possible roles of the annotated genes/proteins. Clustered transcripts were annotated using the homology approach to assign functional annotation using BLAST tool [24] against “Fungi” data from the Uniprot database. Transcripts were assigned with a homolog protein from other organisms if the match was found at e-value less than e^−5^ and minimum similarity greater than 30%. The top 50 highly upregulated transcripts of each GT and CAT were further manually annotated using the Ensembl Fungi database (https://fungi.ensembl.org/Colletotrichum_gloeosporioides_cg_14_gca_000446055/Info/Index (accessed on 14 June 2019) for *C. gloeosporioides* to find out more accurate molecular and biological functions.

#### 2.3.6. Transcription Factors and Secreted Protein Analysis

Assembled transcripts were translated into protein using TransDecoder. The putative transcription factors (TFs) were predicted using the Fungal Transcription Factors Database (http://ftfd.snu.ac.kr (accessed on 4 October 2019). Transcription factor analysis was performed based on homology approach using NCBI-blast program. 

For effector protein identification, proteins with signal peptides were identified using SignalP [25]. Proteins with a transmembrane helix predicted using TMHMM [26] were excluded from the analysis. Candidate effector proteins were identified by subcellular localization prediction using TargetP [27] and WolfPsort [28]. All putative proteins with a SignalP D-score = Y were considered. These proteins were then scanned for transmembrane spanning regions using TMHMM, and proteins with 0 transmembrane domains (TM) were retained. Finally, proteins with location predicted as Loc = S [25] using TargetP and proteins predicted as extracellular (Ext >17) using WolfPSort were retained in the final candidate effector protein dataset. The Pfam search (EMBL-EBI) was performed for functional annotation and to find out the possible biological roles of secreted proteins during GT formation and CAT fusion.

#### 2.3.7. Data Availability

RNA-seq data were submitted to the NCBI SRA database under accession numbers SRR12245378, SRR12245377, and SRR12245376.

### 2.4. Real-Time qRT-PCR Validation

Validation of differential gene expression obtained from the transcriptome analysis was performed using qRT-PCR assay on total cDNA samples from the three life stages, viz., resting conidia, GT, and CAT of *C. gloeosporioides*. For this, 27 differentially expressed genes from resting conidia, GT, and CAT detected by RNA-seq were randomly chosen for validation by qPCR. The Beta-tubulin gene was used as a reference gene for normalization. Specific primer pairs were designed for these 27 selected genes using the Primer3 Software to generate final amplicon sizes of each gene between 50 to 150 bp with 60 °C melting temperature. All primers used in this study are listed in Table S8. Quantitative real-time PCR (qRT-PCR) was carried out as previously described [29]. In brief, total RNA (1 μg) from resting conidia, GTs, and CATs were reverse transcribed to cDNA with an oligo (dT) primer using SuperScript III (Invitrogen, Carlsbad, CA, USA) according to the manufacturer’s instructions. A regular PCR amplification for each primer pair was performed to optimize the annealing temperature. The qRT-PCR was performed in PCRmax Eco 48 Real-Time Cycler using 50 ng of cDNA, 5 μL of SYBR™ Green PCR Master Mix (Applied Biosystems), 10 pmol of sense primers, and 10 pmol of antisense primers in a final volume of 10 μL. The PCR cycle conditions were set as follows: 95 °C for 30 s, followed by 40 cycles of 95 °C for 10 s, 55 °C for 30 s, and 60 °C for 15 s. Lastly, in order to check the amplification of a single targeted amplicon, melting curves were analyzed for all the primer pairs. Expression of genes was evaluated according to their relative quantification using the 2^−∆∆Ct^ method [30]. Each sample was run in triplicates for all selected and reference genes. Data were analyzed using the EcoStudy software v5.2. A correlation between expression levels of DEGs by qRT-PCR and RNA seq was evaluated using log2fold expression values.

### 2.5. Statistical Analyses

The GT formation and CAT fusion data were analyzed by Analysis of Variance (ANOVA) with Tukey’s post hoc test using GraphPad Prism 5 Statistics Software. Differences with a *p*-value < 0.05 were considered statistically significant. All GT formation and CAT fusion assays were performed in triplicates and with 300 conidial numbers.

## 3. Results

### 3.1. CAT Fusion and GT Formation Are Mutually Exclusive in C. gloeosporioides

**Figure 1:**
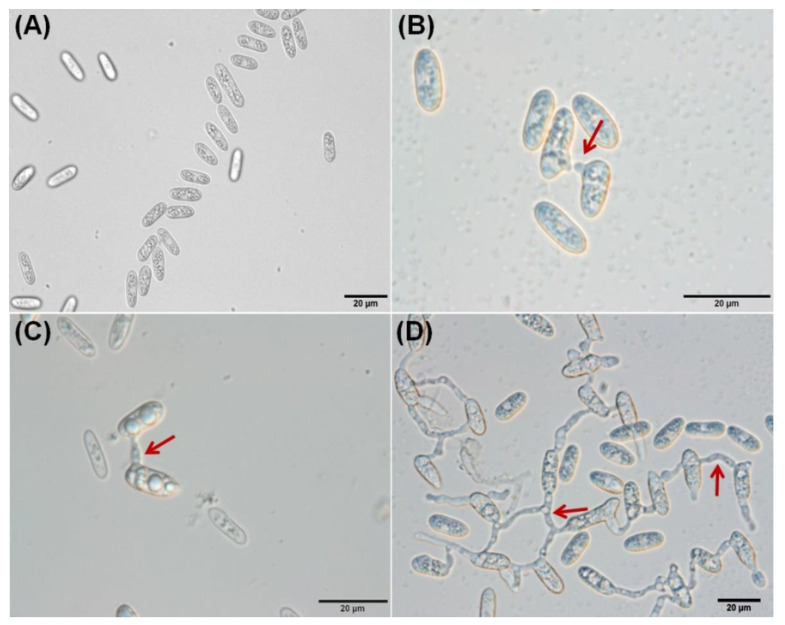
Different stages of CAT fusion in *C. gloeosporioides* in vitro. (**A**) CAT induction. (**B**) CAT homing. (**C**) CAT fusion. (**D**) CAT network. Arrow indicates CAT fusion. Scale Bar = 20 μm.

**Figure 2:**
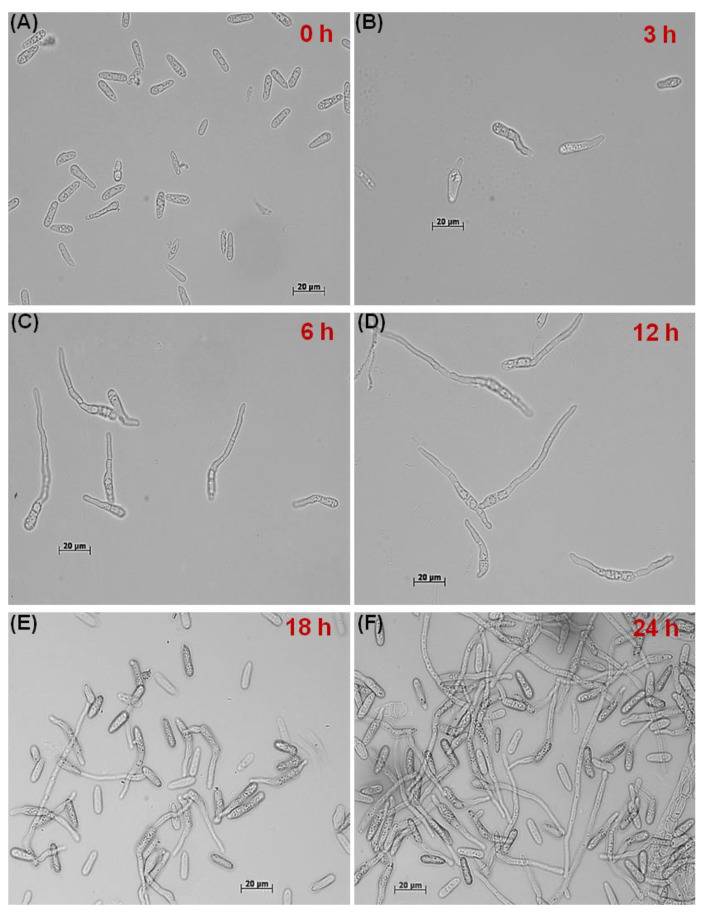
Different stages of GT formation at different time points in *C. gloeosporioides* in vitro. (**A**) Swelling and adhesion of conidia. (**B**) GT initiation. (**C**–**E**) GT elongation. (**F**) Hyphal network. Scale Bar = 20 μm.

The different stages of CAT fusion were demonstrated in *C. gloeosporioides*. Initially, conidia become primed for CAT fusion as a result of CAT induction (Figure 1A). Subsequently, CATs home towards each other, which is known as CAT homing (Figure 1B). Finally, the CATs were fused to each other, constituting the third stage, i.e., CAT fusion (Figure 1C). Later on, with increasing, incubation time, CAT connections expanded and formed a CAT network (Figure 1D) through 72 h of incubation. The different stages of the GT formation were also determined in *C. gloeosporioides*, which could be divided into three stages, namely, conidial swelling and adhesion at 3 h post-incubation (hpi) (Figure 2A,B), GT initiation including polarized growth at 6 hpi (Figure 2B,C), GT elongation with hyphal growth and formation of septa 12–18 hpi (Figure 2D,E), and finally, GT network formation at and after 24 hpi (Figure 2F). 

**Figure 3:**
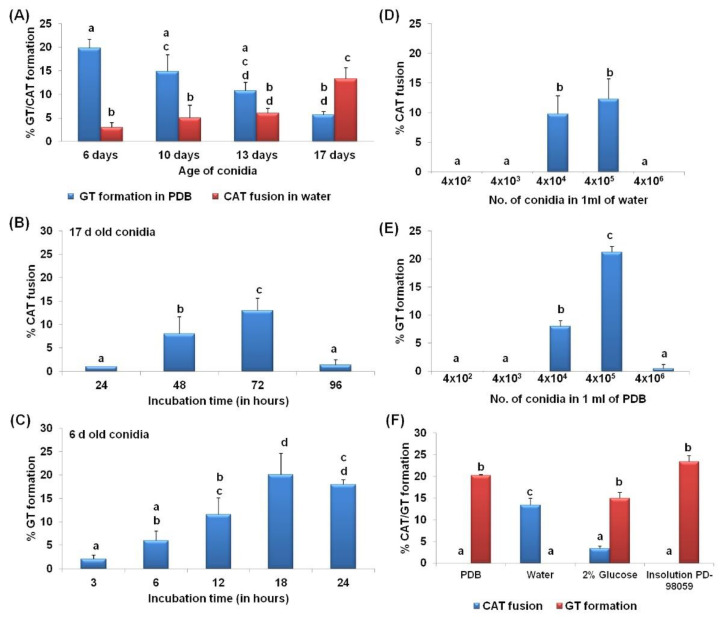
Germ tube formation and conidial anastomosis tube fusion dynamics in *C. gloeosporioides*. (**A**) Percentage GT/CAT formation in different aged conidia, viz., 6, 10, 13, and 17 days of *C. gloeosporioides*. (**B**) Percentage CAT fusion in 17 days old conidia of *C. gloeosporioides* incubated for different incubation times, viz., 24, 48, 72, and 96 h. (**C**) Percentage GT formation in 6 days old conidia of *C. gloeosporioides* incubated for different incubation times, viz., 3, 6, 12, 18, and 24 h. (**D**) Percentage CAT fusion in different conidial densities, viz., 4 × 10^2^, 4 × 10^3^, 4 × 10^4^, 4 × 10^5^, and 4 × 10^6^ of 17-day-old conidia incubated in 1 mL of water. (**E**) Percentage GT formation in different conidial densities, viz., 4 × 10^2^, 4 × 10^3^, 4 × 10^4^, 4 × 10^5^, and 4 × 10^6^ of 6-day-old conidia incubated in 1 mL of PDB. (**F**) CAT and GT formation percentage in PDB, water, glucose, and Insolution^™^ PD98059 (inhibitor of MEK), CAT fusion induced in water supplemented with glucose and Insolution^™^ PD98059; GT formation was assayed in PDB supplemented with glucose and Insolution^™^ PD98059. GT/CAT formation was quantified as the percentage of conidia involved in GT/CAT formation. Average from three replicates (*n* = 3), and 300 conidial numbers were counted per replicate. Bar indicates standard deviation. Statistical significance of differences was analyzed by one-way ANOVA with Tukey’s multiple comparison post-hoc test (bars with the same letter are not significantly different; *p* ≤ 0.05).

When CAT fusion and GT formation was studied in differently aged conidia, it was observed that young conidia (6 days) in PDB were efficient in forming GT, while older conidia (17 days) in sterile distilled water were good at forming CAT (Figure 3A). The younger (6 days) and older (17 days) conidia in PDB do not form CAT at all, while in water, they showed 3 ± 1% and 13.3 ± 2.3% CAT fusion, respectively. As the age of the conidia increases, the GT formation percentage decreases. In contrast, the CAT fusion percentage increased with the increasing age of conidia. The young conidia (6 days) in PDB showed 19.8 ± 1.8% GT formation and no CAT fusion, while the same aged conidia in water showed 3 ± 1% CAT fusion and no GT formation (Figure 3A). The old conidia (17 days), when incubated in water, showed 13.3 ± 2.3% CAT fusion and no GT formation, while the same aged conidia in PDB showed 5.7 ± 0.6% GT formation and no CAT fusion (Figure 3A). To determine the optimal incubation for GT formation, 6-day-old conidia were incubated in PDB for 3, 6, 12, 18, and 24 h; the maximum GT formation (20.1 ± 4.5%) was seen at 18 hpi (Figure 3B). When 17-day-old conidia were incubated in distilled water for 24, 48, 72, and 96 h, the maximum CAT fusion frequency (13.5 ± 2.6%) was observed at 72 hpi (Figure 3C). Therefore, most of the further experiments were conducted using these optimized parameters for in vitro GT and CAT induction. 

### 3.2. CAT Fusion and GT Formation Are Dependent on the Conidial Density

A density of 4 × 10^4^ and 4 × 10^5^ conidia per ml of water was found to be optimal for CAT fusion in *C. gloeosporioides*. The CAT fusion percentages were 9.8 ± 3% and 12.3 ± 3.4% in 4 × 10^4^ and 4 × 10^5^ conidia per ml of water, respectively (Figure 3D). However, for GT formation 4 × 10^5^ conidial density was optimal, resulted in 21.2 ± 1% GT formation (Figure 3E). GT formation decreased to 8 ± 1% when the conidial density reduced to 10-fold, that is 4 × 10^4^ conidia per ml of PDB (Figure 3E). Much lower and higher conidial densities, viz., 4 × 10^2^, 4 × 10^3^, and 4 × 10^6^ were not favorable for GT as well as CAT formation. These results suggest that CAT fusion and GT formation were dependent on the conidial concentration.

### 3.3. Differential Nutritional Requirements for CAT versus GT Formation

The conidia of *C. gloeosporioides* failed to undergo CAT fusion in the presence of nutrient-rich medium such as PDB (Figure 3F). As shown in the previous section, a high percentage of CAT fusion (13.4 ± 1.5%) was observed in sterile distilled water. However, when the conidia were incubated in water with 2% glucose, the CAT fusion frequency decreased to 3.4 ± 0.5% (Figure 3F). Thus, it shows that CAT fusion occurred predominantly during nutrient starvation conditions and was inhibited in high nutrient conditions. Furthermore, the conidia failed to induce CAT fusion in the presence of InSolution^™^ PD98059, an inhibitor of MEK, suggesting the involvement of the MAPK pathway in CAT fusion in *C. gloeosporioides* (Figure 3F). 

On the other hand, GT formation in *C. gloeosporioides* conidia was observed in the presence of nutrients such as PDB and 2% glucose at the frequencies of 20.3 ± 0.2% and 15 ± 1.4%, respectively (Figure 3F). However, GT formation was not seen in nutrient limiting conditions such as water as a medium, indicating that GT formation occurs predominantly in nutrient-rich environments but not in nutrient limiting conditions. Surprisingly, GT formation frequency increased up to 25 ± 1.3% in the InSolution™ PD98059, an MEK inhibitor (Figure 3F). 

### 3.4. RNA-Seq Data Analysis

**Figure 4:**
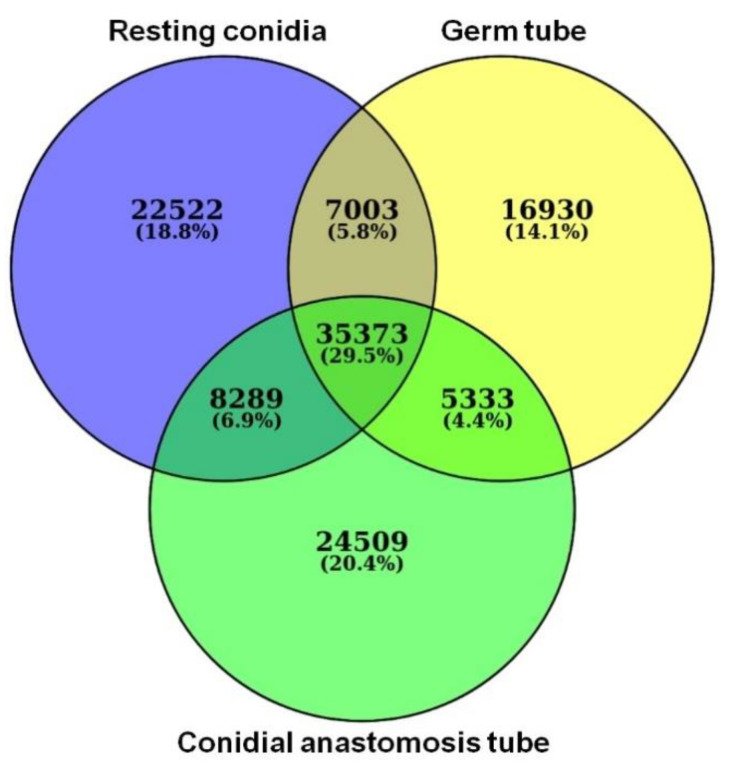
Venn diagram showing the number of transcripts in resting conidia, germ tube, and conidial anastomosis tube.

All RNA samples had an RNA integrity number (RIN) greater than 9.0. An average of 41.62 million raw sequencing PE reads were produced in total for three samples, out of which an average of 39 million reads were used for the downstream analysis after preprocessing. Thus, for every sample on an average of 95% of high-quality data was retained and used for analysis (Table S1). A total of 211,330 transcripts were obtained, which consisted of 73,187, 64,639, and 73,504 transcripts of resting conidia, GT, and CAT, respectively. Among these transcripts, 35,373 were present in all three life stages, while 22,522, 16,930, and 24,509 transcripts were uniquely expressed in resting conidia, GT, and CAT, respectively (Figure 4).

The assembly of high-quality reads was performed separately for resting conidia, GT, and CAT. Sample wise transcripts were further clustered into 121,260 transcripts with an average length of 603 bp and N50 of 1849 bp (Table S2). The length of the final assembly ranged from 37 to 31,011 bps.

### 3.5. Gene Ontology Enrichment Analysis of GT and CAT 

The assembled transcripts for GT and CAT were individually grouped into ten molecular functions (MF) and ten biological processes (BP) (Figure S1). The transcripts, which were significantly enriched and variable in numbers under the biological processes, included fungal type (GO:0031505), carbohydrate (GO:0005975), and transmembrane (GO:0055085); the molecular functions included chitin (GO:0008061), serine-type (GO:0004252), iron (GO:0005506), oxidoreductase (GO:0016491), and hydrolase (GO:0004553) during both GT formation and CAT fusion (Figure S1). The comparison of the biological processes of GT and CAT indicated that fungal type (GO:0031505) GO terms were higher in CAT than in GT. In contrast, the carbohydrate (GO:0005975) GO terms were more significant in GT than in CAT (Figure S1A,B). However, when the molecular functions were compared, it was observed that the serine-type (GO:0004252) and iron (GO:0005506) GO terms were higher in CAT than in GT (Figure S1C,D). 

### 3.6. Identification and Functional Annotation of DEGs in GT and CAT

The DEGs were further analyzed between GT and CAT. The transcripts which show a log2fold change less than −1 are represented as downregulated, the values greater than 1 are defined as upregulated, and between −1 to 1 are termed as neutrally regulated.

**Figure 5:**
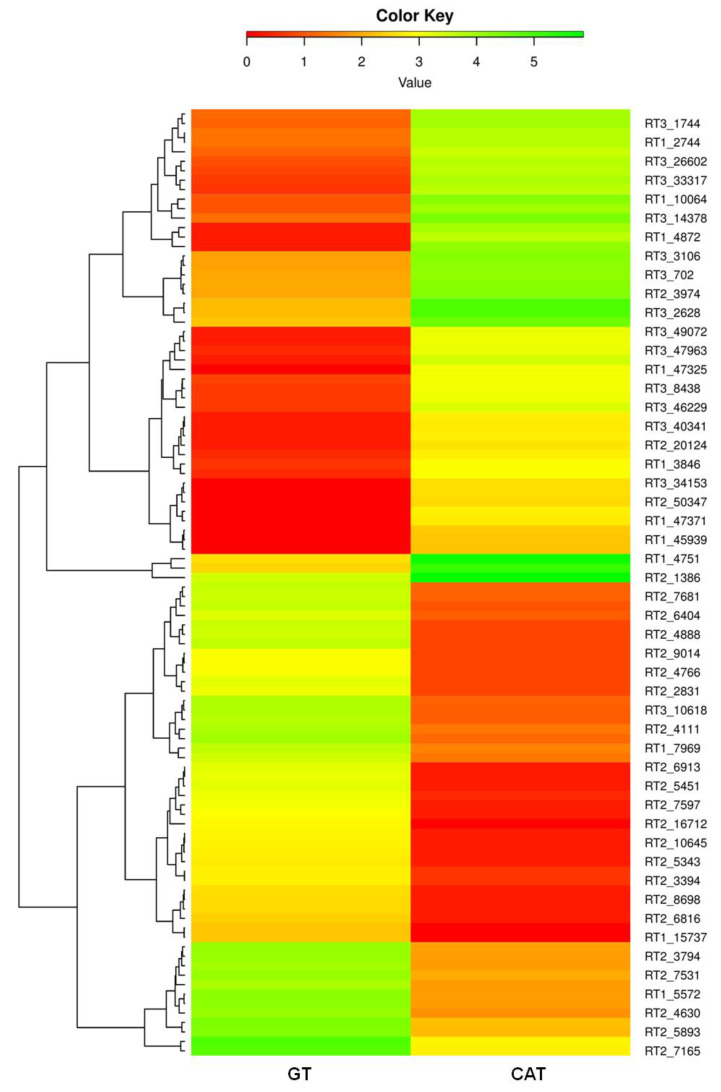
Heatmap depicting differential expression profile of selected genes involved during GT formation versus CAT fusion in *C. gloeosporioides*.

Heatmaps of the top 50 differentially expressed genes in GT and CAT showed a significant variation in their expression profiles. The top 25 upregulated transcripts of GT showed significant downregulation in CAT. On the other hand, the top 25 upregulated genes from CAT were significantly downregulated in GT (Figure 5). Heatmaps based on DEGs of resting conidia v/s GT and resting conidia v/s CAT also showed remarkable differential expression in these life stages of *C. gloeosporioides* (Figure S2). Manual functional annotation of the top 50 transcripts for both GT and CAT resulted in specific categories of proteins, which probably play an important role in the development of CAT and GTs in *C. gloeosporioides* (Tables S3 and S4). Out of these top 50 differentially expressed transcripts, we found four major groups of highly upregulated genes during GT formation, which were involved in cell wall degradation, host-fungus interaction, germination, transport, signaling, and virulence and whose possible functions and expression values in GT and CAT are given in Table 1. Likewise, out of those top 50 DEGs, four major groups of highly upregulated genes involved in CAT fusion were stress response, cell wall, membrane transport, and signaling, cytoskeleton, cell cycle, and cell rescue and pathogenicity genes, whose possible functions and expression values in CAT and GT are given in Table 2.

### 3.7. Transcription Factor Candidates Involved in GT Formation and CAT Fusion

In this study, a total of 49 Transcription factor (TF) families and 1044 TF proteins were predicted using the Fungal Transcription Factors Database; out of theses, 38 TF families (Table 3) and 220 TF proteins (Table S5) were differentially expressed during GT and CAT induction. Twenty-four TF families, e.g., bZIP, C2H2 zinc finger, HMG, Zn2Cys6, etc., were upregulated during CAT fusion compared to GT formation, while 14 TF families such as AraC type, Homeobox, etc., were upregulated in GT formation (Table 3). Among the 220 transcription factor genes, 42 and 82 were uniquely expressed during GT formation and CAT fusion, respectively (Table S5). 

### 3.8. Effector Candidates Secreted during GT Formation and CAT Fusion

GT formation and CAT fusion in *C. gloeosporioides* involve the deployment of secreted effector proteins. Effector candidates are often <200 amino acids in length and cysteine-rich [31]; therefore, we principally focused on small secreted proteins <250 amino acids in length. There were 1567 effector candidates; of these, 776 and 791 were secreted during GT and CAT formation, respectively. Out of 776 GT effector proteins, 102 were uniquely secreted during GT formation (Table S6), and out of 791 CAT effector proteins, 100 were uniquely secreted during CAT fusion (Table S7).

Differentially expressed secretory proteins were manually annotated and categorized in four and three major groups for GT formation and CAT fusion, respectively. Few selected uniquely expressed effector candidates during GT formation include hydrolytic enzymes, adhesion, germination, hyphal development associated proteins, transport, signaling-related proteins, and proteins responsible for virulence. Some examples of these groups of effector proteins uniquely secreted during GT formation and their possible functions are given in Table 4. Likewise, the major effector proteins uniquely secreted during CAT fusion included stress-associated proteins, cell wall, membrane transport, signaling-related proteins, cytoskeleton, cell cycle, and fungal development associated proteins. Some examples of these groups of effector proteins uniquely secreted during CAT fusion and their possible functions are given in Table 5.

### 3.9. Real-Time qPCR Validation of Selected DEGs

**Figure 6:**
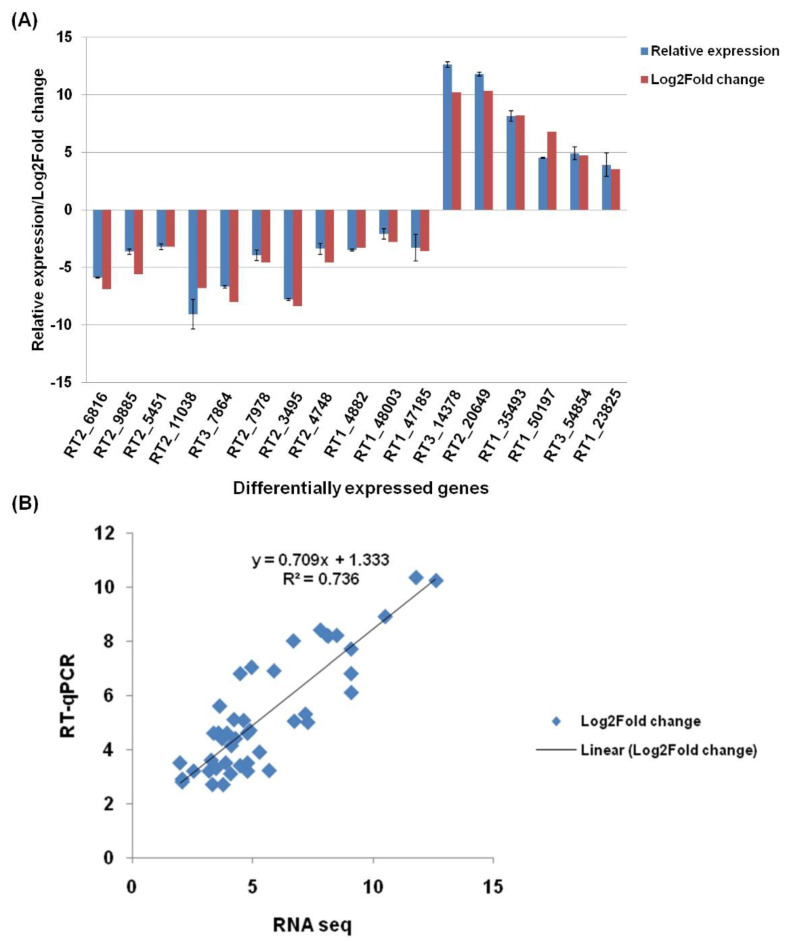
Relative expression of DEGs detected by qPCR and RNA seq. (**A**) Comparison of differential expression of DEGs obtained by qRT–PCR and RNA Seq in GT formation versus CAT fusion in *C. gloeosporioides*. RT1: transcripts of resting conidia, RT2: transcripts of GT, and RT3: transcripts of CAT. (**B**) Scatter plot showing the correlation between expression levels of DEGs detected by qRT–PCR and RNA seq in GT and CAT in *C. gloeosporioides*.

Differentially expressed genes in resting conidia, GT, and CAT identified by RNA-seq were further verified through a quantitative real-time PCR (qRT-PCR). We observed a strong correlation between the qRT-PCR and RNA-Seq analysis of 17 DEGs in GT versus CAT formation (Figure 6A). Similar levels of correlations were also attained in the expression levels of 14 and 13 DEGs in resting conidia v/s GT and resting conidia v/s CAT, respectively (Figure S3). Log2fold expression values were used to evaluate the correlation between RNA-seq and qRT-PCR. The results obtained by qRT-PCR showed significant correlation (R^2^ = 0.736; *p* < 0.01) with transcriptome Log2fold data (Figure 6B). It showed that the RNA-seq experiment was conducted with accuracy and RNA-seq data is reliable to understand the molecular mechanism underlying CAT formation further. 

## 4. Discussion

### 4.1. Differential Physiological Requirements for GT and CAT 

In the present study, we have attempted to understand the required in vitro conditions for GT and CAT formation in *C. gloeosporioides*. We observed significant CAT fusion in 17-day-old conidia and GT formation in 6-day-old conidia (Figure 3A). Our results seem to fit with the conidial age requirement of the genus *Colletotrichum*; older conidia (17 d old) for CAT fusion and younger conidia (6 d old) for GT formation were preferred in *C. gloeosporioides* (Figure 3A). It has been reported that CAT fusion is dependent on conidial age for different species; 16-day-old conidia of *C. lindemuthianum* and *C. gossypii* were more appropriate for CAT fusion, while 20-day-old conidia of *C. fructicola* and *C. nymphaeae* were suitable for CAT fusion [6,8,14]. In contrast, the younger conidia (10 d old) of *C. lindemuthianum* were suitable for GT formation in nutrient-rich medium such as PDB [10]. However, the conidial age requirements for both CAT fusion and GT formation in *F. oxysporum* and *N. crassa* were 7–10 and 5–6 days, respectively [4,5,9]. The GT/CAT formation was shown to be conidial density-dependent in *N. crassa* and *F. oxysporum* [4,5]. In these fungi, the conidial number threshold requirement for CAT induction was more or less same, i.e., 1 × 10^6^ conidia/mL [4,5,6,8,9,32]. The GT induction has also been shown to depend on conidial density in *F. oxysporum;* the maximum GT formation was observed in 1 × 10^5^ conidia per mL [4]. The conidial number requirement for CAT fusion and GT formation in *C. gloeosporioides* was also found to be 4 × 10^5^ conidia/mL (Figure 3D,E). It indicates that a conidial threshold is a requisite for the CAT induction as well as GT formation (Figure 3D,E). 

We observed that the conidia of *C. gloeosporioides* could efficiently form CAT in the absence of nutrients (water) compared to nutrient-rich conditions such as PDB. The presence of glucose in water also reduces CAT fusion percentage in this fungus (Figure 3F). On the other hand, significant GT formation occurred only in the presence of nutrients such as PDB, and nutrient limiting conditions negatively affected GT formation (Figure 3F). These observations indicate that the CAT fusion occurs in nutrient limiting conditions; conversely, GT formation occurs only in the nutrient-rich conditions in *C. gloeosporioides*. It has been shown previously that nutrient limiting conditions induce CAT fusion, and nutrient-rich conditions promote GT formation in few fungi [4,10]. In another species of the genus *Colletotrichum,* i.e., *C. lindemuthianum*, and *C. graminicola,* CAT fusion does not occur in nutrient-rich conditions such as PDB/oatmeal agar (OMA) and even in nutrient-poor Vogel’s medium, while it occurs only in water media [10,12]. Meanwhile, in other CAT-forming fungi such as *N. crassa* and *F. oxysporum*, CAT fusion occurs in the presence of nutrients, e.g., Vogel’s medium for *N. crassa* and YNB+KNO3/1% PDB +NaNO3 for *F. oxysporum* [4,5,9,33,34]. We have also shown that CAT fusion in *C. gloeosporioides* is inhibited by a MAP kinase inhibitor InSolution^™^ PD98059 (Figure 3F), suggesting a possible role of MAP kinase pathway in CAT fusion in *C. gloeosporioides*. Interestingly, in the presence of the MAP kinase pathway inhibitor InSolution^™^ PD98059, the frequency of GT formation increased considerably compared to PDB (Figure 3F), thereby suggesting that this pathway may not be a significant player in early GT formation. Since there are different MAPK pathways in fungal pathogens, it might be possible that a different MAPK pathway is operational during GT formation. However, the MAPK pathway was found to be essential in appressorium formation in the same fungus [35]. 

We propose that the choice of conidia to either form GT or CAT depends upon multiple factors, viz., conidial age (internal nutrients), availability of external nutrients, and/or starvation stress conditions. We have shown that the older conidia were superlative for CAT fusion while younger conidia were ideal for GT formation. We believe that increased CAT fusion reduces the internal resources for GT formation; however, the conidia’s decision to either form CAT or GT does not only rely solely upon the internal resources. We had shown that when the older conidia (17 days) were incubated in water (no nutrients), they could undergo extensive CAT fusion and no GT formation. However, when the same aged older conidia were incubated in PDB, they could also form GT but at a reduced rate compared to younger conidia (6 and 10 days old) (Figure 3A). In *C. graminicola*, the older conidia were shown to undergo autophagic activity due to nutrient stress [36]. Therefore, we hypothesize that as the age of conidia increases, the internal resources get depleted; in this scenario, if the external resources are provided, then conidia could still form GTs to a very lesser extent. On the contrary, in the absence of external nutritional resources, the older conidia undergo extensive CAT fusion to exchange their nutrients for survival. 

After understanding the differential physiological requirements of CAT and GT formation, we attempted to decipher the differential molecular prerequisites of these two processes in *C. gloeosporioides* by undertaking RNA-sequencing and analysis. As a result, we observed significant differences in transcriptomes of GT formation and CAT fusion. Furthermore, we identified many differentially expressed genes, transcription factors, and secretory effector proteins during GT formation and CAT fusion in *C. gloeosporioides*. 

### 4.2. Transcriptome Analysis of GT 

Genes involved in cell wall degradation, host-fungus interaction, germination, transport, signaling, and virulence were found to be upregulated during GT formation. Previously, it has been shown that genes responsible for plant cell wall degradation, secondary metabolism, and detoxification were upregulated during GT formation in *C. fructicola* [37]. We also observed that some hydrolytic enzymes involved in plant cell wall degradation such as pectin lyase, glycosyl hydrolase families, mandelate racemase/muconate lactonizing enzyme, and fungal cellulose-binding domain-containing protein were upregulated during GT formation (Table 1). In the present study, Hydrophobin 2 and HsbA genes were also upregulated during GT formation in *C. gloeosporioides*, which are probably involved in conidial adhesion. Fungal hydrophobins are small, secreted hydrophobic proteins involved in the adhesion of conidia to the host surface prior to GT formation [38]. Fungal hydrophobin, HsbA-like protein were shown to be upregulated during GT and appressorium development in *A. oryzae* and *C. fructicola* [37,39]. Other significant DEGs include GPI-anchored cell wall beta-1,3-endoglucanase Egl, which probably plays a role in germination, and hydrophobic surface binding protein A, which increases the hydrophobicity of conidia, aerial hyphae, and fruiting bodies.

Fungal transporter proteins play a broad range of biological functions and an essential role in the pathogenicity of phytopathogens [40,41,42]. In the present study, few transporters and signaling genes such as Fg-gap repeat protein, phosphate-repressible phosphate transporter, transmembrane amino acid transporter, MFS monosaccharide transporter, high-affinity methionine permease, and Uso1/p115-like vesicle tethering protein were upregulated during GT formation (Table 1). These transports and associated signaling genes are probably important to sense the favorable conditions for germination, which results in GT formation. However, in previous studies, it has been demonstrated that an ABC protein CgABCF2 was required for appressorium formation and plant infection in *C. gloeosporioides* [35,43].

Some secondary metabolites and virulence associated genes were shown to add to pathogenicity in *C. gloeosporioides* [44]. We have also found that various secondary metabolite backbone genes such as peptidase S41 family protein, nrps-like protein, FAD-dependent oxidoreductase, and cyanide hydratase were upregulated during GT formation, which may contribute to pathogenicity [45,46]. During GT formation, few other highly upregulated genes such as polysaccharide deacetylase, FGGY-family pentulose kinase, acetylornithine aminotransferase, GMC oxidoreductase, GNAT family acetyltransferase, CAP-22 protein, and NmrA family transcriptional regulator may also be implicated for pathogenicity in *C. gloeosporioides* (Table 1). 

Transcription factors play important roles in various biological processes. Out of 220 annotated TF genes, 42 were uniquely expressed during GT formation, and these genes are probably involved in the regulation of GT formation in *C. gloeosporioides* (Table S5). Effector proteins are usually secretory proteins, which can play an important role in plant infection. For example, one study showed that effectors are secreted from appressorium before host invasion in *C. higginsianum*, a close relative of *C. gloeosporioides* [47]. In our study, we have identified uniquely expressed effector candidate genes during GT formation. Interestingly, the annotated effectors could also be categorized in the same categories, which were based on DGE analysis of GT formation. The uniquely expressed effector gene candidates include hydrolytic enzymes, adhesion, germination, hyphal development associated proteins, transport, signaling related proteins, and proteins responsible for virulence (Table 4). Transcripts of gene glucanases that degrade glucan in the fungal cell wall for cell wall modulation were observed during germination in *Aspergillus niger* [2]. Various transcripts and secretory effectors of glucanases were observed in our study during GT formation, which might be involved in the cell wall morphogenesis leading to germination. Trehalose accumulates in dormant conidia of *A. niger* and is degraded during germination [2]. We detected trehalase as a secretory effector during GT formation, which probably provides energy by degrading trehalose in *C. gloeosporioides*. Other noteworthy uniquely expressed effector gene candidates include carbonic anhydrase and manganese lipoxygenase, which might be important for fruiting body development, ascospore germination, and accelerating programmed spore germination, respectively (Table 4). 

The evidences for the involvement of MAPK in conidial germination are conflicting and equally diverse between the species. The deletion of MAPK gene CMK1 in *C. lagenarium* prevents germination, whereas deletion of MAPK gene pmk1 in *Magnaporthe grisea* blocks appressorium development but not germination [1]. The MAPK pathway was shown to be essential for appressorium development in *C. gloeosporioides* [35]. However, in our case, GT formation was not inhibited in the presence of MAPK pathway inhibitor Insolution™ PD98059, and no transcripts related to MAPK pathway could be detected in GT transcriptome, thereby indicating that MAPK pathway may not be essential for early GT formation in *C. gloeosporioides*.

### 4.3. Transcriptome Analyses of CAT

The CAT fusion seems to involve a different set of genes compared to GT formation while retaining few common transcripts in both of these processes. We observed that the major groups of highly upregulated genes involved in CAT fusion belong to stress response, cell wall integrity, membrane transport, signaling, cytoskeleton, cell cycle, and cell rescue processes (Table 2).

In this study, we found many stress-associated genes that were upregulated during CAT fusion such as pyridine nucleotide-disulfide, formate dehydrogenase, NADH dehydrogenase, oxalate decarboxylase family bicupin, maleylacetate reductase, O-acetyl homoserine amino carboxypropyl transferase, iron-sulfur cluster assembly protein 1, bZIP transcription factor, HhH-GPD superfamily base excision DNA repair protein, ribulose-phosphate 3-epimerase, and dipeptidase A. All these genes are believed to be involved in oxidative stress response. Reactive oxygen species (ROS) are known to play an important role in redox signaling pathways, including cell differentiation, development, and cytoskeleton remodeling [48]. NADPH-oxidases (NOX) are responsible for the production of superoxide (ROS) by oxidizing NADPH and reducing molecular oxygen, which is believed to be involved during communication, cell fusion, and CAT fusion in several fungi, e.g., *N. crassa, F. oxysporum,* and *V. dahliae* [48,49,50,51]. It has been shown that NADPH oxidase BcNoxA or Nox regulator BcNoxR gene deletion mutant of Grey mold *Botrytis cinerea* were defective in CAT fusion [52]. In our study, NADPH oxidase cytochrome p450 and stress response regulator SrrA, which probably participate in redox signaling and stress signal transduction, were also upregulated during CAT fusion (Table 2). It has been previously documented that starvation response is correlated with the expression of genes encoding oxidative stress response in *S. cerevisiae* [53]. Further oxidative stress was shown during the aging of stationary cultures of *S. cerevisiae* [54]. Our study demonstrated that the older conidia under nutritional starvation exhibit efficient CAT fusion, and our transcriptome data analysis also revealed high expression of oxidative stress related genes. This suggests that starvation in *C. gloeosporioides* conidia induced a strong oxidative response, and the genes involved in this response might play important roles in CAT fusion in this fungus. 

The CAT fusion involves sensing of quorum through signaling and transport followed by cell wall and cell membrane remodeling. Many membrane transporters and cell wall modulation genes, including MFS transporter, transmembrane amino acid transporter, peptidoglycan-N-acetylglucosamine deacetylase, and Glycosyltransferase family 2 protein, were found to be upregulated during CAT fusion. In addition, other important transcripts, viz., alcohol dehydrogenase GroES-like domain, flippase-like domain-containing protein, and acetyl-CoA carboxylase, were upregulated during CAT fusion, which probably play roles in protein folding, pheromone response, cytoskeletal dynamics, cell division, lipid metabolism, and fatty acid synthesis in *C. gloeosporioides*. 

The cytoskeleton actin is important for several functions, including cell polarity, exocytosis, endocytosis, septation, movement of organelle, and chemotropic growth of fungi [48]. In *N. crassa*, the actin cables and patches are significantly increased at CAT tips during chemotropic interactions between germlings or conidia [55]. However, microtubules are dispensable for germling communication, while actin is critically important in *N. crassa* [55]. We observed that the MreB/Mbl proteins, which are constituents of the eukaryotic actin cytoskeleton, were highly upregulated during CAT fusion in *C. gloeosporioides*, suggesting that actin is critically essential for polarized growth and CAT fusion. We have previously shown that an inter-specific CAT fusion between *C. gloeosporioides* and *C. siamense* involved nuclear transfer [11]. Therefore, we assume that even in the present study, CAT fusion in *C. gloeosporioides* also involves nuclear exchange. Corroborating to this belief, we have observed that several cell cycle and cell rescue genes were highly upregulated during CAT fusion in *C. gloeosporioides*, which include the F-box domain gene, which is thought to be involved in mitosis or cell division cycle; DNA repair; and the MutS domain V gene which is probably involved in the protection of cells from DNA damage-induced genome instability by repairing the DNA mismatch. 

Transcription factors play important roles in various biological processes. Out of the total 220 annotated TF genes, 82 were uniquely expressed during CAT fusion, and these genes are probably involved in the regulation of CAT fusion in *C. gloeosporioides* (Table S5). Two such transcription factors, adv-1 and pp-1, a C2H2-Zn2C transcription factor, were previously shown to be necessary for germling communication and fusion in *N. crassa* [56,57]. Similarly, we have also observed that few transcription factors, including C2H2-Zn2C, Zn 2cys6, bZIP transcription factors, were upregulated during CAT fusion in *C. gloeosporioides*.

We have also found various unique effector genes specifically secreted during CAT fusion, and interestingly the major transcripts could be grouped in similar categories such as general DEGs, which included stress-associated proteins, cell wall, membrane transport, signaling related proteins, cytoskeleton, cell cycle, and fungal development-associated proteins. We observed a few stress-associated effector genes, including Acetyl-CoA acetyl transferase, Calcineurin-like phosphoesterase, LipA, NB-ARC domain-containing protein, and WSC domain-containing protein-coding gene, which might be involved in the physiological stress response and nutrient availability signals during CAT fusion in *C. gloeosporioides*. The WSC domain-containing protein-coding gene has previously been shown to be important in germling and hyphal fusion in *N. crassa* [58]. We observed few MAPK pathway-related transcripts as secretory effectors during CAT fusion, which included Hkr1p, which is known to regulate the high-osmolarity glycerol (HOG) and filamentous growth (FG) MAPK pathway; the PAN domain, which is essential for RasA-mediated morphogenetic signaling; and Pmp3, which is involved in mating and cell wall integrity MAPK pathway. The upregulation of MAP kinase pathway genes corroborates well with our physiological data on inhibition of CAT fusion in the presence of InSolution™ PD 98059, an inhibitor of MEK. These data together indicate that the MAPK pathway is probably involved in CAT fusion in *C. gloeosporioides*. Calcium and some calcium-dependent genes have been shown to play a critical role during the membrane fusion in germling communication in various fungi [48]. Calcium is also essential for polarized hyphal growth and fusion in *N. crassa* [59,60]. We detected some calcium-dependent genes as secretory effectors, which included calcium influx-promoting protein ehs1, calcium-related spray gene, SOCE-associated regulatory factor of calcium homoeostasis gene, which are probably involved in maintaining cell wall integrity, calcium signaling, calcium stress response, growth, and virulence during CAT fusion in *C. gloeosporioides*. We also detected some transcripts of cell division cycle genes, which uniquely secreted during CAT fusion, viz., coronin, which is known to play a role in the organization and dynamics of actin and F-actin remodeling, and Wlm domain-containing genes, which is associated with sister chromatid separation and segregation during mitosis.

## 5. Conclusions

**Figure 7:**
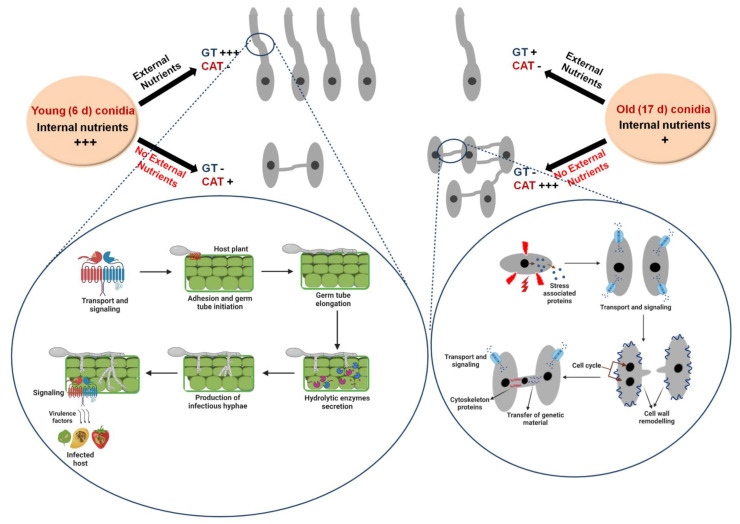
Model representing the differential physiological and molecular requirements of GT formation and CAT fusion in *C. gloeosporioides* (Model created with BioRender.com).

We propose a model to explain the mutual exclusiveness of GT formation and CAT fusion and their dependency on conidial age, availability of external nutrients, and differential RNA profiles (Figure 7). The younger conidia (6 days) are expected to have substantial internal resources; therefore, when such young conidia are incubated in a nutrient-rich medium, they tend to form GT extensively, and when these young conidia are incubated in a nutrient-poor medium, they do not form GT and negligible CAT. On the other hand, when the older conidia (17 days) are believed to be exhausted of internal nutrients, and therefore, when incubated in a nutrient-rich medium, they could form GT with lower frequency. However, when such older conidia are incubated in water (no nutrients), they tend to form extensive CATs to exchange the nutrients for survival (Figure 7). During the GT formation, genes responsible for adhesion, GT elongation, and germination were highly upregulated, which signifies that they tend to undergo GT formation under suitable conditions when the host plant is available for the conidia by infectious hyphae. The transcriptome data also revealed high expression of genes coding for hydrolytic enzymes, infectious hyphae, and virulence factors during GT formation, which again signifies that eventually, conidia will have to degrade the plant cell wall and establish an infection in the plant, which also requires certain virulence factors. On the contrary, during the CAT fusion, stress response genes were expressed to cope with the stress. Further, for CAT fusion, extensive transport, signaling, and cell wall remodeling are required; therefore, genes coding for these processes were found to be highly expressed during CAT fusion. Since CAT fusion involved forming a bridging tube and transferring cell organelles, including nuclei, many gene coding for cytoskeleton and cell cycle regulation are expressed uniquely during CAT fusion. To conclude, we have systematically addressed the relationship between availability of nutrients, and conidial age with respect to CAT and GT formation, and deciphered that these two processes are mutually exclusive. The first ever whole transcriptome comparison of CAT and GT revealed significant differences in their gene expression profiles in *C. gloeosporioides*. It would be interesting to know whether other fungal species capable of forming CAT also exhibit similar transcriptome profiles. 

## Figures and Tables

**Table 1:** Selected upregulated genes during GT formation showing their possible functions and respective expression values in GT and CAT in *C. gloeosporioides*.

Transcript ID	Gene Name	Possible Functions	GT	CAT
Cell wall degrading enzymes				
RT2_5451	Pectin lyase	Degrades pectin	**1712**	**2**
RT2_4111	Glycosyl hydrolase family 76	Degrades cellulose, hemicellulose, and lignin found in plant cell wall	**7920**	**24**
RT2_6404	Mandelate racemase/muconate lactonizing enzyme domain-containing protein	Involved in the breakdown of lignin-derived aromatics	**1848**	**13**
RT2_6816	Fungal cellulose binding domain-containing protein	Active in plant cell-wall hydrolysis	**254**	**2**
Host-fungus interaction and germination				
RT2_16712	Quinone reductase	Host-fungus interaction & infection	**761**	**1**
RT1_10305	Hydrophobin 2	Involved in adhesion	**9563**	**16**
RT1_4917	Cerato-platanin	Role during fungus-plant interactions and infection	**16,867**	**62**
RT2_5893	GPI-anchored cell wall beta-1,3-endoglucanase EglC	Role in germination	**21,332**	**143**
RT3_7789	Hydrophobic surface binding protein A	Increase the hydrophobicity of conidia, aerial hyphae, and fruiting bodies	**86,745**	**621**
Transport and signaling				
RT2_6913	Fg-gap repeat protein	Important for ligand binding	**1571**	**2**
RT2_4144	Phosphate-repressible phosphate permease/transporter	Membrane transport proteins	**1556**	**2**
RT2_4587	Transmembrane amino acid transporter	Ubiquitin dependent endocytosis and signaling responses	**3406**	**6**
RT1_15737	MFS monosaccharide transporter	Membrane transport proteins	**182**	**1**
RT2_4766	High affinity methionine permease	Cysteine and methionine transport across plasma membrane	**1027**	**6**
RT3_9669	Uso1/p115 like vesicle tethering protein	Intracellular protein transport	**24,227**	**173**
Virulence				
RT2_3663	Peptidase S41 family protein	Involved in pathogenesis	**4430**	**6**
RT2_27038	nrps-like protein	Involved in virulence	**1299**	**3**
RT2_3495	Polysaccharide deacetylase	Involved in virulence	**3561**	**10**
RT2_10908	FGGY-family pentulose kinase	Involved in virulence	**625**	**2**
RT1_5572	Acetylornithine aminotransferase	Required for growth, conidiogenesis, and pathogenicity	**17,456**	**64**
RT2_5240	GMC oxidoreductase	Role in pathogenicity	**324**	**2**
RT2_2131	GNAT family acetyltransferase	Role in chitin metabolism of fungi development and pathogenicity	**10,433**	**68**
RT2_7531	CAP-22 protein	Expressed in the conidium during appressorium formation	**13,700**	**100**
RT1_7969	NmrA family transcriptional regulator	Required for the invasive virulence	**4225**	**33**
RT1_7368	Cyanide hydratase	Catalyzes the hydration of cyanide to formamide. It may be necessary for plant pathogenic fungi in infection of cyanogenic plants	**2902**	**23**

Different shades of colors (white, grey and black) represent expression of GT and CAT 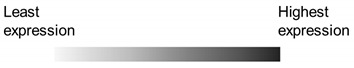.

**Table 2:** Selected upregulated genes during CAT fusion, their possible functions, and respective expression values in CAT and GT in *C. gloeosporioides*.

Transcript ID	Gene Name	Possible Function	CAT	GT
Stress associated genes				
RT1_6087	Taurine catabolism dioxygenase	Utilization of taurine under sulfate starvation	**8376**	**2**
RT3_33317	Pyridine nucleotide-disulfide	Involved in cellular oxidative stress response	**7093**	**5**
RT3_14378	Formate dehydrogenase	Role under hypoxia stress response	**24,057**	**20**
RT3_26602	NADH dehydrogenase	Role in oxidative stress	**8132**	**14**
RT1_5411	Oxalate decarboxylase family bicupin	Role in stress response	**8913**	**17**
RT3_3106	Maleylacetate reductase	Has an oxidoreductase activity	**19,971**	**75**
RT1_51991	Proline dehydrogenase	Role in osmotic, drought, and salinity stress	**213**	**1**
RT1_52730	O-acetyl homo serine amino carboxypropyl transferase	Involved in oxidative stress	**773**	**14**
RT1_51711	Iron sulfur cluster assembly protein 1	Involved in oxidative stress	**79**	**2**
RT1_51144	bZIP transcription factor	Responses to oxidative stress	**266**	**21**
RT1_59783	HhH-GPD superfamily base excision DNA repair protein	Involve in stress and hormone signaling	**24**	**2**
RT1_47091	Ribulose-phosphate 3-epimerase	Cellular response to oxidative stress	**34**	**3**
RT3_1744	NADPH cytochrome p450	Role in redox signaling	**5805**	**8**
RT1_54531	Stress response regulator SrrA	Involved in stress signal transduction	**62**	**2**
RT1_20193	Dipeptidase A	Role in fungal growth and heat shock stress	**13**	**2**
Cell wall, membrane transport, and signaling				
RT1_11804	MFS transporter	Membrane transport proteins	**14,522**	**2**
RT1_871	Transmembrane amino acid transporter	Promoting endocytosis and triggering signaling responses	**19,654**	**95**
RT3_59479	Peptidoglycan-N-acetylglucosamine deacetylase	Involved in cell wall morphogenesis and remodeling	**201**	**6**
RT1_49642	Alcohol dehydrogenase GroES-like domain	Functions in protein folding and intercellular signaling	**59**	**2**
RT3_50435	Flippase-like domain-containing protein	Pheromone response, cytoskeletal dynamics, cell division, lipid metabolism, and lipid signaling	**76**	**3**
RT3_62264	Acetyl-CoA carboxylase biotin carboxylase subunit	Fatty acid synthesis pathway	**20**	**1**
RT3_59549	Glycosyltransferase family 2 protein	Cell wall synthesis and other functions	**79**	**5**
Cytoskeleton, cell cycle, and cell rescue				
RT1_7001	MreB/Mbl protein	Constituents of the eukaryotic cytoskeleton (tubulin, actin)	**2907**	**2**
RT3_59288	F-box domain protein	Involved in cell division cycle, glucose sensing, and stress response	**1332**	**2**
RT3_43400	DNA repair protein	Protects cells from DNA damage-induced genome instability	**597**	**1**
RT1_35493	Zn 2cys6 transcription factor	Involved in meiosis, stress response, and pleiotropic drug resistance	**582**	**2**
RT3_50458	MutS domain V	DNA mismatch repair protein	**562**	**3**
RT1_51248	Actin	Major components of the cytoskeleton	**4419**	**131**
Pathogenicity associated proteins				
RT1_2872	Tetraspanin 10	Small integral membrane proteins, required for pathogenicity	**17,772**	**71**
RT3_8438	Integral membrane protein	Required for pathogenicity	**1046**	**5**

Different shades of colors (white, grey and black) represent expression of GT and CAT 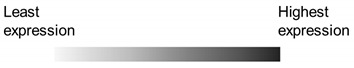.

**Table 3:** Transcription factors families and their expression values involved in GT formation and CAT fusion in *C. gloeosporioides*.

TF Family Name	Number of TF Families	
	GT Formation	CAT Fusion
APSES	**16**	**14**
AraC type	**509**	**443**
AT-rich interaction region	**8**	**13**
BED-type predicted	**4**	**2**
bHLH	**35**	**38**
Bromodomain transcription factor	**9**	**8**
bZIP	**109**	**130**
C2H2 zinc finger	**590**	**665**
CCHC-type	**142**	**179**
DHHC-type	**34**	**43**
Forkhead	**9**	**15**
GATA type zinc finger	**54**	**53**
Grainyhead/CP2	**5**	**4**
GRF-type	**12**	**7**
Helix-turn-helix type 3	**3**	**4**
HMG	**228**	**264**
Homeobox	**24**	**16**
Homeodomain-like	**221**	**226**
HTH	**1**	**2**
LSD1-type	**13**	**18**
MADS-box	**31**	**27**
MIZ-type	**13**	**10**
Myb	**60**	**62**
NDT80/PhoG like DNA-binding	**4**	**5**
Negative transcriptional regulator	**7**	**8**
NF-X1-type	**8**	**17**
Not1	**7**	**10**
OB-fold	**500**	**540**
p53-like transcription factor	**9**	**12**
Psq	**6**	**4**
Rad18-type putative	**28**	**30**
ssDNA-binding transcriptional regulator	**16**	**20**
TEA/ATTS	**20**	**24**
Transcription factor jumonji	**9**	**12**
Transcription factor TFIIS	**13**	**11**
Tubby transcription factors	**10**	**9**
UAF complex subunit Rrn10	**3**	**4**
Zn2Cys6	**2057**	**2134**

Different shades of colors (white, grey and black) represent expression of GT and CAT 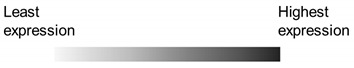.

**Table 4:** Uniquely expressed secretory effector proteins during GT formation in *C. gloeosporioides*.

Transcript ID	Protein Names	Possible Functions
Hydrolytic enzymes		
RT2_4955.p1	Acetyl esterase	Degradation of hemicelluloses and pectin
RT2_12928.p2	Alkaline phosphatase	Role in P mobilization from organic substrates under P starvation conditions
RT2_9649.p1	Amidohydrolase	Type of hydrolase that acts upon amide bonds
RT2_4824.p1	Berberine bridge enzyme	It is a cellobiose oxidase that degrades the carboxymethylcellulose (CMC), xylan, and lignin
RT2_14227.p1	Beta-1,3-endoglucanase	Role in cell wall softening
RT2_10729.p1	Endo-chitosanase	Role in degradation of the deacetylated portion of chitin in the fungal cell wall
RT2_9596.p1	Extracellular cellulase	Degrades cellulose and some other related polysaccharides
RT2_1942.p1	Glycosyl hydrolase family 15	Degrades cellulose, hemicellulose, and lignin found in plant cell wall
RT2_15512.p2	Glycosyl hydrolase family 28	Degrades cellulose, hemicellulose, and lignin found in plant cell wall
RT2_618.p1	Glycosyl hydrolase family 31	Degrades cellulose, hemicellulose, and lignin found in plant cell wall
RT2_12327.p1	Xylosidase arabinofuranosidase	Role in xylan degradation
Adhesion, germination, and hyphal development		
RT2_1963.p1	Acid trehalase	Provides energy during conidial germination
RT2_6726.p1	Carbonic anhydrase	Role in fruiting body development and ascospore germination
RT2_2892.p2	Endoglucanase 3	Role in germination
RT2_10852.p2	Fungal hydrophobin	Role in adhesion
RT2_5434.p1	Glycosyltransferase sugar-binding region containing DXD motif	Role in pathogenesis of plants by enabling hyphal growth
RT2_4975.p1	Manganese lipoxygenase	Accelerates programmed spore germination
RT2_13809.p1	Pollen proteins Ole e I like	Potentially implicated in pollen germination
RT2_18566.p1	Spindle poison sensitivity protein	Role as spindle machinery for chromosome segregation and cytokinesis
RT2_10806.p1	WD40 domain protein beta propeller	Regulates fungal cell differentiation
RT2_11269.p2	Acid phosphatase, putative	Involved in fungal growth
RT2_4368.p1	Class III aminotransferase, putative	Regulate the fungus-host interaction
Transport and signaling		
RT2_12723.p2	Bacterial extracellular solute-binding protein, family 3	Act as chemoreceptors, recognition constituents of transport systems, and initiators of signal transduction pathways
RT2_18223.p1	Eukaryotic porin	It is an important regulator of Ca2+ transport in and out of the mitochondria
RT2_1739.p1	WSC domain-containing protein	Responses to stress cues and metal ions
Virulence		
RT2_4851.p1	Calcineurin-like phosphoesterase	Role in stress survival, sexual differentiation, and virulence
RT2_11269.p2	Phosphoinositide phospholipase C, Ca^2+^-dependent	Roles in growth, stress tolerance, sexual development, and virulence
RT2_3804.p1	Secretory lipase, putative	Potential virulence factors
RT2_5435.p1	Versicolorin b synthase	Role in the biosynthesis of aflatoxins
RT2_15735.p1	Zinc-binding dehydrogenase	Role in infection

**Table 5:** Uniquely expressed secretory effector proteins during CAT fusion in *C. gloeosporioides*.

Transcript ID	Protein Names	Possible Functions
Stress associated		
RT3_6147_m.31990	Acetyl-CoA acetyltransferase	Role in oxidative and cell wall stresses
RT3_8826_m.37297	Calcineurin-like phosphoesterase	Involved in stress survival and fungal virulence
RT3_10144_m.39239	Chitin synthesis regulation, resistance to Congo red	Role in cell wall stress, noxious chemicals, and osmotic pressure changes
RT3_12971_m.42487	Cytochrome oxidase assembly protein	Cox11p is an integral protein of the inner mitochondrial membrane that is essential for cytochrome c oxidase assembly. Role in response to hydrogen peroxide exposure
RT3_12332_m.41840	LipA and NB-ARC domain-containing protein	Function as key integrators of stress and nutrient availability signals
RT3_571_m.6736	Pyridoxamine 5’-phosphate oxidase	Role in oxidative stress
RT3_15296_m.44520	Spherulation-specific family 4	Required for spherulation under starvation conditions
RT3_2386_m.18522	SWIM zinc finger	Roles in the stress response and virulence
RT3_10404_m.39608	WSC domain-containing protein	Responses to stress cues and metal ions
Cell wall, membrane transport, and signaling		
RT3_17141_m.45950	Ankyrin-3-like protein 3	Roles in cell motility, activation, proliferation, contact, and the maintenance of specialized membrane domains
RT3_870_m.9203	Aspartic endopeptidase	Role in cell-wall assembly, remodeling, and cell wall integrity
RT3_2825_m.20686	Blastomyces yeast-phase-specific protein	Required for cell wall morphogenesis and pathogenesis
RT3_100_m.1747	Cytochrome p450	Role in redox signaling
RT3_6853_m.33623	Endo-beta-1,6-glucanase	Cell wall morphogenesis and remodeling
RT3_3953_m.25310	Gamma-glutamyltranspeptidase	Role in the vacuolar transport and metabolism of glutathione
RT3_2987_m.21396	GPI biosynthesis protein family Pig-F	Required for Cell Wall Biogenesis and Normal Hyphal Growth
RT3_25_m.564	Hkr1p	Regulate the filamentation and mitogen-activated protein kinase pathway
RT3_2892_m.20978	Mid2 like cell wall stress sensor	Required for stress-induced nuclear to cytoplasmic translocation of Cyclin C and cell wall integrity signaling pathway
RT3_18063_m.46545	MIP family channel protein	Involved in the transport of water and neutral solutes across the membranes
RT3_268_m.3768	NCS1 nucleoside transporter	Responsible for uptake of purines, pyrimidines, or related metabolites
RT3_704_m.7811	PAN domain	Essential for RasA-mediated morphogenetic signaling
RT3_5718_m.30849	Pectin esterase	Facilitate plant cell wall modification and subsequent breakdown
RT3_18612_m.46914	PQ loop repeat	Function as cargo receptors in vesicle transport
RT3_11944_m.41457	Probable lipid transfer	Involved in lipid transfer
RT3_21269_m.48652	Protein serine/threonine kinase	Roles in cellular function such as regulation of signaling pathways
RT3_3541_m.23752	Protein YOP1	Involved in membrane/vesicle trafficking
RT3_543_m.6478	Proteolipid membrane potential modulator	Pmp3 is transcriptionally regulated by the Spc1 stress MAPK (mitogen-activated protein kinases) pathway
RT3_2326_m.18214	SOCE-associated regulatory factor of calcium homoeostasis	Negative regulator of intracellular calcium signaling
RT3_2348_m.18321	Stretch-activated Ca2+-permeable channel component	Required for Ca2+ influx induced by the mating pheromone, alpha-factor
Cytoskeleton, cell cycle, and fungal development		
RT3_12912_m.42432	Alkaline proteinase	Role in fungal physiology and development
RT3_20705_m.48270	Coronin	Role in the organization and dynamics of actin, F-actin remodeling
RT3_13797_m.43265	Frequency clock protein	Regulates various aspects of the circadian clock in Neurospora crassa
RT3_16749_m.45643	Galactoside-binding lectin	Role in fruiting body development
RT3_15066_m.44317	Nucleoside diphosphate kinase	Regulation of spore and sclerotia development
RT3_1_m.4	WD40 domain-containing protein	Regulates fungal cell differentiation
RT3_3897_m.25109	Wlm domain-containing protein (Fragment)	Related to the DNA damage response proteins WSS1 involved in sister chromatid separation and segregation

## Data Availability

RNA-seq data were available at NCBI SRA database under accession numbers SRR12245378, SRR12245377, and SRR12245376.

## Supplementary Materials

The following are available online at https://www.mdpi.com/article/10.3390/jof7070509/s1. Figure S1: Gene ontology enrichment of biological processes and molecular functions during GT and CAT fusion in *C. gloeosporioides.* (A,B) GO counts of highly enriched GO biological processes terms in GT and CAT, respectively. (C,D) GO counts of highly enriched GO molecular function terms in GT and CAT, respectively. Figure S2: Heatmaps of the differential expression profile of selected genes between (A) Resting conidia versus GT formation. (B) Resting conidia versus CAT fusion. Figure S3: A graph of the level of correlation between relative expression obtained by qRT-PCR and RNA-Seq log2fold change of (A) 14 DEGs in resting conidia versus GT formation. (B) 13 DEGs in resting conidia versus CAT fusion. RT1: transcripts of resting conidia, RT2: transcripts of GT, and RT3: transcripts of CAT. Table S1: Read statistics of prepared libraries of resting conidia, GT, and CAT. Table S2: Assembly statistics of prepared libraries of resting conidia, GT, and CAT. Table S3: Top 50 upregulated genes during GT formation. Table S4: Top 50 upregulated genes during CAT fusion. Table S5: Differentially expressed transcription factor proteins involved in GT formation and CAT fusion. Table S6: Uniquely expressed effector candidate proteins during GT formation. Table S7: Uniquely expressed effector candidate proteins during CAT fusion. Table S8: List of primers used in the study for real time qRT-PCR.
