# Supplementary material for: Differential Physiological Prerequisites and Gene Expression Profiles of Conidial Anastomosis Tube and Germ Tube Formation in Colletotrichum gloeosporioides
Source: J Fungi (Basel). 2021 Jun 25;7(7):509. doi: 10.3390/jof7070509 (PMC8306183; doi:10.3390/jof7070509)
Supplement: Supplementary file 1 [file jof-07-00509-s001.zip › Supplementary data/Table S5.pdf]

Table S5: Differentially expressed transcription factor proteins involved in GT formation and CAT fusion.

S. N.	Protein names	Number of proteins	
		GT	CAT
1	2-dehydro-3-deoxygluconokinase (EC 2.7.1.45)	1	0
2	2-isopropylmalate synthase	0	1
3	30S ribosomal protein S12	1	4
4	3'-5'-exoribonuclease	1	0
5	3-beta hydroxysteroid dehydrogenase/isomerase family protein	21	16
6	3-demethylubiquinone-9 3-methyltransferase	0	1
7	3-hydroxyacyl-CoA dehydrogenase, putative	4	9
8	40S ribosomal protein S11-A (RP41) (S18) (Small ribosomal subunit protein uS17-A) (YS12)	0	1
9	4-hydroxyphenylpyruvate dioxygenase	0	1
10	50S ribosomal protein L19	0	1
11	54S ribosomal protein rml2, mitochondrial (L2)	0	1
12	60S ribosomal protein L2	0	2
13	60S ribosomal protein L2-C (K37) (K5) (KD4)	0	1
14	Ab1-133	1	0
15	ABC transporter ATP-binding protein (Duplicated ATPase domains)	18	21
16	Acetyl-CoA C-acetyltransferase	0	1
17	Acetyl-coenzyme A synthetase (Fragment)	5	7
18	Acyl-CoA dehydrogenase	0	3
19	Acyl-CoA dehydrogenase (Acyl-CoA dehydrogenase, N-terminal domain protein) (Butyryl-CoA dehydrogenase) (EC 1.3.8.1)	12	20
20	Acyl-CoA hydrolase (Acyl-CoA thioesterase) (Thioesterase superfamily protein)	2	0
21	Alcohol dehydrogenase (Cytochrome c)	0	3
22	Aliphatic sulfonates import ATP-binding protein SsuB (EC 3.6.3.-)	0	2
23	Alpha/beta hydrolase (Fragment)	0	1
24	Alpha-L-fucosidase	1	3
25	Arabinan endo-1,5-alpha-L-arabinosidase	8	4
26	Arginine--tRNA ligase (EC 6.1.1.19) (Arginyl-tRNA synthetase) (ArgRS)	0	1
27	Argininosuccinate synthase (EC 6.3.4.5) (Citrulline--aspartate ligase)	3	6
28	Asparagine-rich zinc-finger protein	2	0
29	Asparagine--tRNA ligase, cytoplasmic (EC 6.1.1.22) (Asparaginyl-tRNA synthetase) (AsnRS)	0	1
30	Aspartate--tRNA ligase, cytoplasmic (EC 6.1.1.12) (Aspartyl-tRNA synthetase) (AspRS)	1	0
31	Aspartate-tRNA(Asn) ligase	1	0
32	Aspartyl-tRNA synthetase	8	13
33	Aspyridones cluster regulator apdR (Aspyridones biosynthesis protein R)	0	1
34	ATPase (Fragment)	0	1
35	Binuclear zinc transcription factor	11	6
36	Branchpoint-bridging protein (Mud synthetic-lethal 5 protein) (Splicing	0	1

	factor 1) (Zinc finger protein BBP)		
37	BZIP transcription factor	2	5
38	BZIP transcription factor (AtfA), putative	0	4
39	C2H2 zinc finger protein	2	0
40	C6 finger domain protein	3	1
41	C6 transcription factor (Gal4), putative	0	2
42	C6 transcription factor (UaY), putative	1	0
43	C6 transcription factor QutA, putative	3	1
44	C6 transcription factor RosA-like, putative	0	1
45	C6 transcription factor RosA	0	1
46	C6 transcription factor, putative	123	138
47	C6 zinc finger protein	0	2
48	Calcium-translocating P-type ATPase, PMCA-type	19	24
49	Carbohydrate kinase family protein (Fragment)	0	1
50	Carboxylic ester hydrolase (EC 3.1.1.-)	56	44
51	Casein kinase II subunit beta (CK II beta)	0	1
52	Cell pattern formation-associated protein stuA	3	1
53	Choline dehydrogenase (Fragment)	22	12
54	Chorismate synthase (CS) (EC 4.2.3.5) (5-enolpyruvylshikimate-3-phosphate phospholyase)	1	4
55	Class 2 transcription repressor NC2	0	2
56	Conserved hypothetical membrane protein	1	3
57	Conserved hypothetical secreted protein	0	1
58	Cullin binding protein CanA, putative	1	3
59	Cullin-1	3	8
60	Cutinase transcription factor 1 beta	9	6
61	Cyanate hydratase (Cyanase) (EC 4.2.1.104) (Cyanate hydrolase) (Cyanate lyase)	1	3
62	Cystathionine gamma-synthase (Fragment)	9	4
63	Cytochrome c oxidase polypeptide 4 (EC 1.9.3.1) (Cytochrome aa3 subunit 4) (Cytochrome c oxidase polypeptide IV)	0	1
64	Cytoplasmic 60S subunit biogenesis factor REI1 (Required for isotropic bud growth protein 1) (pre-60S factor REI1)	1	0
65	Cytoplasmic asparaginyl-tRNA synthetase	1	4
66	DEHA2A12694p	0	1
67	DEHA2D14718p	0	1
68	DEHA2E11682p	0	4
69	DEHA2G20328p	0	1
70	Deoxyribonuclease HsdR	0	1
71	Developmental regulator FlbA	2	0
72	Dihydroxyacetone kinase DhaK subunit	5	3
73	Dihydroxy-acid dehydratase (DAD) (EC 4.2.1.9)	8	5
74	DNA polymerase III subunit alpha (EC 2.7.7.7)	1	5
75	DNA replication licensing factor mcm2 (EC 3.6.4.12) (Cell division control protein 19) (Minichromosome maintenance protein 2)	3	1
76	DNA replication licensing factor mcm5	1	0
77	DNA replication licensing factor MCM6 (EC 3.6.4.12)	1	0

	(Minichromosome maintenance protein 6)		
78	DNA-binding protein HEXBP	9	4
79	Endo-1,4-beta-glucanase, putative	8	11
80	Eukaryotic translation initiation factor 5A-1 (eIF-5A-1)	2	0
81	Eukaryotic translation initiation factor 5A-2 (eIF-5A-2)	0	2
82	Exosome complex component rrp40 (Ribosomal RNA-processing protein 40)	0	1
83	Exosome complex exonuclease RRP4	3	1
84	FAD-dependent oxidoreductase	1	0
85	Ferrous iron transport protein B	0	1
86	FKH1 transcription factor-like protein	0	2
87	Fructose-1,6-bisphosphate aldolase (Fructose-1,6-bisphosphate aldolase, class II) (Fructose-bisphosphate aldolase) (Fructose-bisphosphate aldolase class II) (EC 4.1.2.13)	4	1
88	Fungal specific transcription factor domain protein	0	3
89	Fused acetyl/propionyl-CoA carboxylase subunit alpha/methylmalonyl-CoA decarboxylase subunit alpha	10	14
90	Galactoside O-acetyltransferase	4	2
91	Gamma-glutamyltranspeptidase (EC 2.3.2.2)	3	7
92	Heat shock transcription factor	0	1
93	Heavy metal translocating P-type ATPase	8	13
94	Helicase	8	11
95	Helix-loop-helix DNA-binding domain-containing protein	2	0
96	High-affinity nicotinic acid transporter	72	92
97	High-copy mep suppressor	0	1
98	Homeobox domain-containing protein	2	0
99	Homeobox transcription factor phx1	0	1
100	Homeobox transcription factor, putative	3	0
101	Homoserine kinase (HK) (HSK) (EC 2.7.1.39)	1	0
102	Hydrophobe/amphiphile efflux-1 (HAE1) family transporter	0	2
103	Imidazolonepropionase (EC 3.5.2.7) (Imidazolone-5-propionate hydrolase)	0	3
104	Inosine-5'-monophosphate dehydrogenase (IMP dehydrogenase) (IMPD) (IMPDH) (EC 1.1.1.205)	2	6
105	Involucrin repeat protein	1	2
106	Iron-sulfur protein	1	0
107	Isocitrate dehydrogenase [NADP] (EC 1.1.1.42)	4	8
108	J protein JJJ1	0	1
109	Jmjc domain-containing histone demethylase	0	1
110	J-protein (Type III)	0	1
111	Kinesin-like protein bimC	0	2
112	Leptomycin B resistance protein pmd1	49	56
113	Leucine dehydrogenase (EC 1.4.1.9)	1	0
114	Leucyl/phenylalanyl-tRNA--protein transferase (EC 2.3.2.6) (L/F-transferase) (Leucyltransferase) (Phenyalanyltransferase)	0	1
115	Lid2 complex component lid2	1	3
116	Lysine biosynthesis regulatory protein LYS14	1	0

117	Lysine--tRNA ligase (EC 6.1.1.6) (Lysyl-tRNA synthetase)	11	15
118	Malate dehydrogenase (EC 1.1.1.37)	2	0
119	MeaB protein	1	3
120	Membrane dipeptidase (Peptidase family M19)	0	2
121	Methionine gamma-lyase	3	7
122	Methionyl-tRNA formyltransferase (EC 2.1.2.9)	5	1
123	Methylmalonyl-CoA carboxyltransferase	4	1
124	Methylmalonyl-CoA mutase (Fragment)	0	2
125	Minichromosome maintenance-related protein	1	0
126	Mis12-Mtw1 family protein	1	0
127	MIZ zinc finger protein	7	3
128	Molecular chaperone DnaJ	13	19
129	Multiprotein-bridging factor 1	1	0
130	MYB family conidiophore development protein FlbD	0	1
131	Myb-like DNA-binding domain protein	0	1
132	N-acetyldiaminopimelate deacetylase (EC 3.5.1.47)	1	0
133	Negative regulator of pleiotropic drug resistance STB5	0	1
134	NF-X1 finger and helicase domain protein, putative	2	7
135	NF-X1 finger transcription factor, putative	0	3
136	Nicotinate dehydrogenase small FeS subunit (EC 1.17.1.5)	0	3
137	Nodulation protein L	4	1
138	Non-histone chromosomal protein	1	0
139	Nonribosomal peptide synthase, putative	78	14
140	Nucleic binding protein	1	0
141	Nucleolar protein NOP2	5	7
142	O-acetylhomoserineaminocarboxypropyltransferase (EC 2.5.1.49)	5	9
143	Oligopeptide transporter family protein	1	0
144	O-methyltransferase	9	6
145	Origin recognition complex subunit 4	1	4
146	Outer membrane efflux protein	1	3
147	Oxidoreductase	23	25
148	Oxidoreductase, 2OG-Fe(II) oxygenase family	15	19
149	Palmitoyltransferase (EC 2.3.1.225)	22	29
150	Pfs, NACHT and WD domain protein (EC 2.4.2.-)	430	380
151	Phosphate transport system permease protein	0	1
152	Phosphatidylserine decarboxylase proenzyme 2, mitochondrial (EC 4.1.1.65) [Cleaved into: Phosphatidylserine decarboxylase 2 beta chain; Phosphatidylserine decarboxylase 2 alpha chain]	3	5
153	Phosphonates import ATP-binding protein PhnC (EC 7.3.2.2)	0	1
154	Phosphoribosylaminoimidazolesuccinocarboxamide synthase (Fragment)	1	0
155	Polyadenylation factor subunit CstF64, putative	0	1
156	Polyribonucleotide nucleotidyltransferase (EC 2.7.7.8) (Polynucleotide phosphorylase) (PNPase)	0	2
157	Potential protein lysine methyltransferase SET5 (EC 2.1.1.-) (SET domain-containing protein 5)	0	1
158	Pre-mRNA-splicing ATP-dependent RNA helicase PRP28	15	26

159	Probable E3 ubiquitin-protein ligase HUL4 (EC 2.3.2.26) (HECT ubiquitin ligase 4) (HECT-type E3 ubiquitin transferase HUL4)	1	4
160	Proteasome regulatory particle subunit Rpt6, putative	16	19
161	Proteasome-activating nucleotidase	5	7
162	PUT3-like fungal specific transcription factor, putative	0	4
163	Putative histone demethylase JARID1D	0	2
164	Putative methyl-accepting chemotaxis protein	0	1
165	Putative multiprotein-bridging factor 1	2	4
166	Putative NAD(P) transhydrogenase alpha subunit (EC 1.6.1.2)	1	0
167	Pyrophosphate--fructose 6-phosphate 1-phosphotransferase (EC 2.7.1.90) (6-phosphofructokinase, pyrophosphate dependent) (PPi-dependent phosphofructokinase) (PPi-PFK) (Pyrophosphate-dependent 6-phosphofructose-1-kinase)	1	0
168	Quinic acid utilization activator	1	0
169	Regulatory protein alcR (Fragment)	0	1
170	Replication protein A subunit	2	4
171	Replicative DNA helicase (EC 3.6.4.12)	0	1
172	Retaining alpha-galactosidase (EC 3.2.1.22)	0	1
173	RhoGAP and Fes/CIP4 domain protein	2	0
174	Rho-GTPase-activating protein 8	1	3
175	Riboflavin biosynthesis protein RibBA [Includes: 3,4-dihydroxy-2-butanone 4-phosphate synthase (DHBP synthase) (EC 4.1.99.12); GTP cyclohydrolase-2 (EC 3.5.4.25) (GTP cyclohydrolase II)]	5	3
176	Ribosomal protein S12 methylthiotransferase RimO (S12 MTTase) (S12 methylthiotransferase) (EC 2.8.4.4) (Ribosomal protein S12 (aspartate-C(3))-methylthiotransferase) (Ribosome maturation factor RimO)	0	1
177	Ribosomal protein S23 (S12)	1	0
178	Rubredoxin	0	1
179	Sensory transduction protein regX3	1	2
180	Short chain dehydrogenase	6	9
181	Short-chain dehydrogenase, putative	3	6
182	Siderophore transcription factor SreA	4	2
183	Sigma-54 modulation protein	0	1
184	Soluble hydrogenase 42 kDa subunit (EC 1.12.-.-)	1	3
185	Specific RNA polymerase II transcription factor	0	2
186	Stage IV sporulation protein A (EC 3.6.1.3) (Coat morphogenetic protein SpoIVA)	0	1
187	Tagatose-6-phosphate kinase (EC 2.7.1.144)	0	1
188	telomere-associated protein 1	0	1
189	TetR family transcriptional regulator	0	2
190	Thiamine repressible genes regulatory protein thi5 (Transcription factor ntf1 5)	1	0
191	Thiamine ABC transporter permease (Fragment)	0	5
192	TonB-dependent outer membrane receptor	0	1
193	Transcription activator of gluconeogenesis acuK (Acetate non-utilizing mutant protein K)	0	1

194	Transcription activator of gluconeogenesis BDCG_02812	0	1
195	Transcription elongation factor GreA (Transcript cleavage factor GreA)	0	1
196	Transcription factor AbaA	1	0
197	Transcription regulatory protein SNF2 (EC 3.6.4.-) (ATP-dependent helicase SNF2) (Regulatory protein GAM1) (Regulatory protein SWI2) (SWI/SNF complex component SNF2) (Transcription factor TYE3)	2	0
198	Transcriptional activator hac1	2	5
199	Transcriptional activator HAP2	0	1
200	Transcriptional activator of proteases prtT (Zn(2)-C6 zinc finger-containing protein prtT)	3	0
201	Transcriptional activator protein acu-15	1	5
202	Transcriptional activator xlnR	1	4
203	Transport system permease protein	0	2
204	tRNA dimethylallyltransferase (EC 2.5.1.75)	3	1
205	tRNA pseudouridine synthase B (EC 5.4.99.25) (tRNA pseudouridine(55) synthase) (Psi55 synthase) (tRNA pseudouridylate synthase) (tRNA-uridine isomerase)	2	4
206	Two-component system response regulator	2	6
207	Ubiquitin ligase subunit CulD	2	6
208	Ubiquitinyl hydrolase 1 (EC 3.4.19.12)	6	4
209	Uncharacterized FAD-linked oxidoreductase yvdP (EC 1.21.-.-)	28	20
210	UPF0276 protein BN444_02599	0	1
211	UV radiation resistance protein (UVRAG), putative	4	2
212	Vacuolar segregation protein PEP7	1	0
213	Voltage-gated chloride channel (ClcA), putative	8	14
214	Zinc finger protein 32	8	3
215	Zinc finger protein 58	13	24
216	Zinc finger protein GIS2	6	9
217	Zinc knuckle domain containing protein	3	6
218	Zinc knuckle transcription factor (CnjB)	2	6
219	Zinc-responsiveness transcriptional activator	4	0
220	Zn(II)2Cys6 transcription factor	2	4
