# Supplementary figures and images for: Differential Physiological Prerequisites and Gene Expression Profiles of Conidial Anastomosis Tube and Germ Tube Formation in Colletotrichum gloeosporioides
Source: J Fungi (Basel). 2021 Jun 25;7(7):509. doi: 10.3390/jof7070509 (PMC8306183; doi:10.3390/jof7070509)

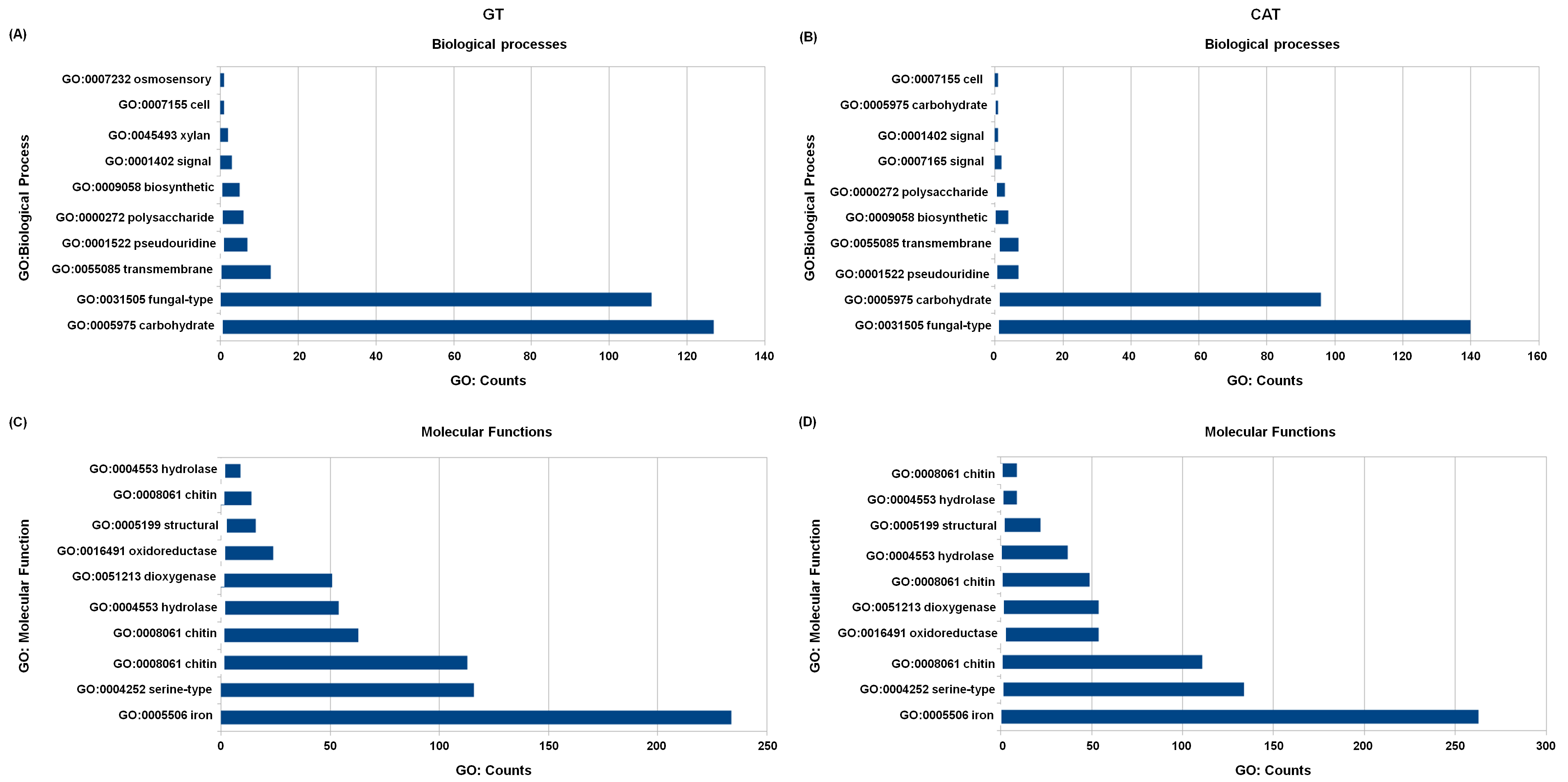

Supplement: Supplementary file 1 [file jof-07-00509-s001.zip › Supplementary data/Supplementary Fig S1.jpg]

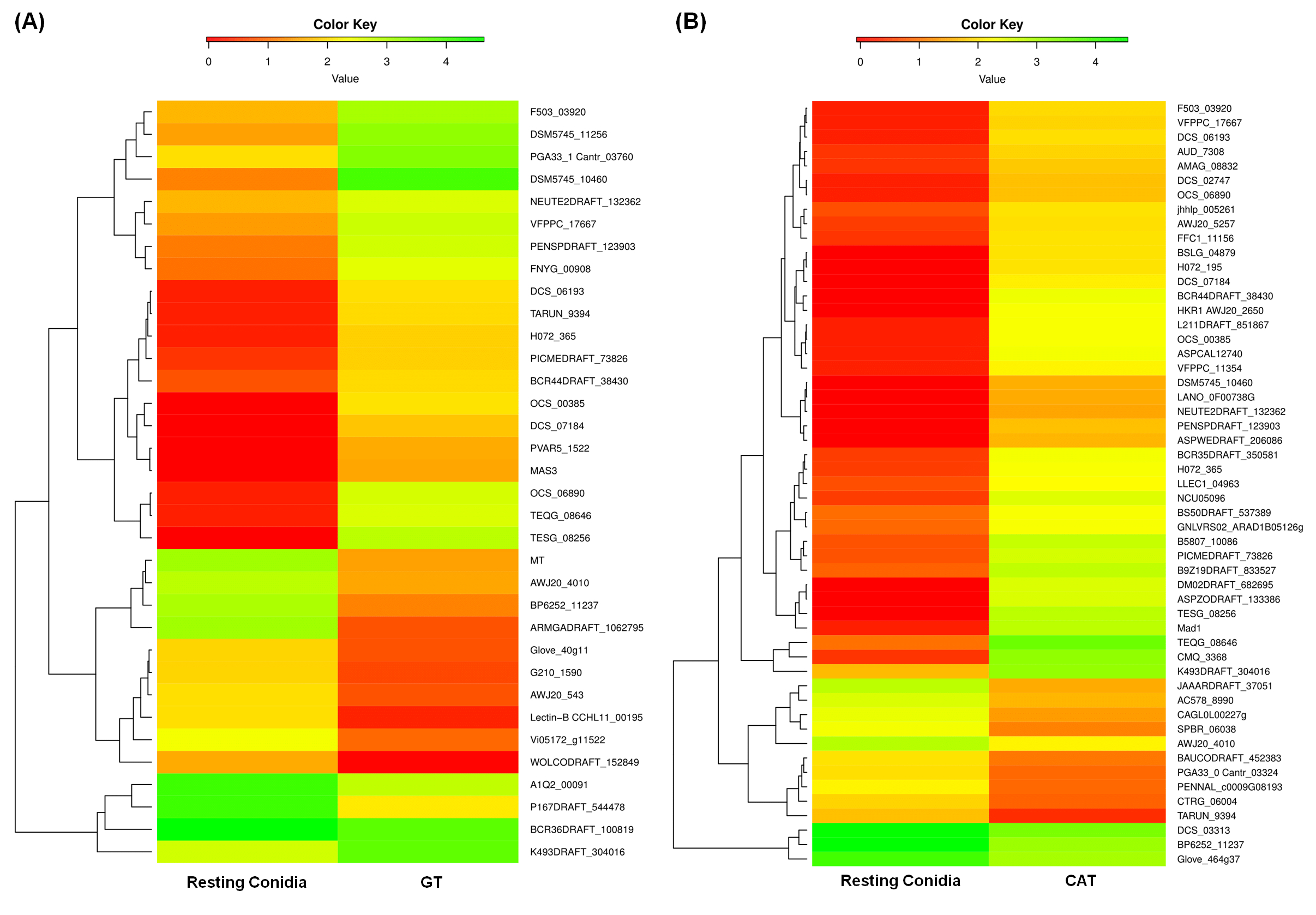

Supplement: Supplementary file 1 [file jof-07-00509-s001.zip › Supplementary data/Supplementary Fig S2.jpg]

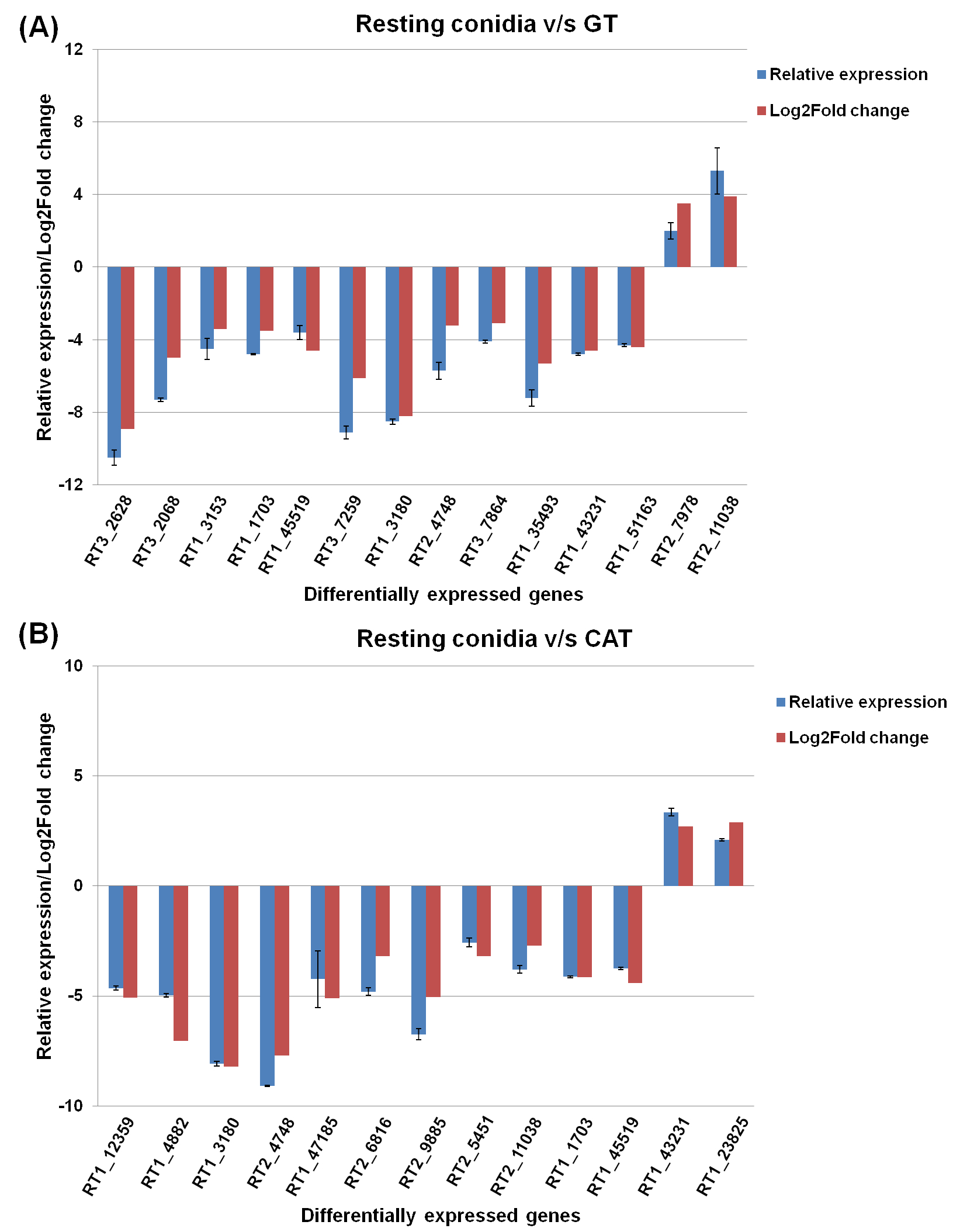

Supplement: Supplementary file 1 [file jof-07-00509-s001.zip › Supplementary data/Supplementary Fig S3.jpg]
